# Global prevalence, site-specific patterns, and key risk factors for osteoporosis and bone loss in systemic lupus erythematosus: a systematic review and meta-analysis

**DOI:** 10.3389/fimmu.2026.1778825

**Published:** 2026-04-15

**Authors:** Chunhui Yu, Jipeng Hui, Siyuan Ding, Xilan Ma

**Affiliations:** 1 The First Clinical College, Liaoning University of Traditional Chinese Medicine, Shenyang, China; 2 The Center for Preventive Treatment of Disease, The Second Affiliated Hospital of Liaoning University of Traditional Chinese Medicine, Shenyang, China; 3 School of Basic Medicine, Liaoning University of Traditional Chinese Medicine, Shenyang, China; 4 The Second Clinical College, Liaoning University of Traditional Chinese Medicine, Shenyang, China; 5 The Third Department of Orthopedics, The Second Affiliated Hospital of Liaoning University of Traditional Chinese Medicine, Shenyang, China

**Keywords:** glucocorticoids, meta-analysis, osteoporosis, prevalence, risk factors, systemic lupus erythematosus

## Abstract

**Background:**

Systemic lupus erythematosus (SLE) is a complex autoimmune disease in which patients have a significantly increased risk of developing osteoporosis (OP) and osteopenia. Despite numerous studies, the global burden of SLE-related OP, its regional distribution patterns and its major risk factors remain poorly quantified and subject to controversy due to heterogeneity in sample sizes, diagnostic criteria and methodologies. To address these gaps in the evidence, we conducted a systematic assessment of the prevalence and risk factors for OP and osteopenia in patients with SLE.

**Methods:**

We conducted a systematic review and meta-analysis. We performed a comprehensive search of Chinese and English databases, including PubMed, Embase, the Cochrane Library, Web of Science, CNKI and WANFANG, up to 26 September 2025. We included observational studies that met the diagnostic criteria for SLE and reported the prevalence of OP or reduced bone mass, as well as associated risk factors. Two reviewers independently conducted literature screening, data extraction and quality assessment. Statistical analysis was performed using Stata 12.0 software; random-effects or fixed-effects models were employed to pool prevalence rates and odds ratios, and subgroup analysis, meta-regression and sensitivity analysis were used to explore sources of heterogeneity.

**Results:**

A total of 59 studies were included. Meta-analysis revealed an overall prevalence of osteoporosis in SLE patients of 16.70% (95% CI: 14.2%, 19.3%) and a prevalence of osteopenia of 39.50% (95% CI: 35.5%, 43.5%). Site-specific analysis indicated that the lumbar spine was the site with the highest prevalence of osteoporosis (10.0%), whilst the femoral neck was the site most commonly affected by osteopenia (44.1%). Subgroup analysis identified several high-risk populations; the prevalence of osteoporosis in postmenopausal women (34.0%) was significantly higher than in premenopausal women (11.6%). Risk factor analysis indicated that advanced age (>50 years, OR = 21.92), long-term glucocorticoid use (OR = 1.63) and prolonged duration of SLE (OR = 1.05) were significant risk factors for OP. Glucocorticoid dosage was positively correlated with risk, with a significant increase in risk observed at daily doses >10 mg.

**Conclusion:**

Patients with SLE are at high risk of osteoporosis and osteopenia; postmenopausal women, Asian patients and those on long-term glucocorticoid therapy should be prioritized for screening and intervention. This study has revealed site-specific patterns of skeletal involvement and quantified the impact of key risk factors. In clinical practice, priority should be given to combined bone density monitoring, focusing on the lumbar spine and femoral neck, in high-risk populations. Furthermore, risk-stratified, proactive bone health management strategies should be implemented, with the aim of shifting the focus from ‘treating fractures’ to ‘preventing fractures’, thereby improving long-term patient outcomes.

**Systematic Review Registration:**

https://inplasy.com/inplasy-2025-12-0043/, identifier INPLASY2025120043.

## Introduction

Systemic Lupus Erythematosus (SLE) is a complex multisystem autoimmune disorder characterized by significant global variations in its incidence and epidemiological features. Studies reveal pronounced disparities in the incidence and prevalence of SLE across different genders, ethnicities, and age groups. The incidence rate is notably higher in women compared to men, particularly among those of reproductive age, a phenomenon that may be attributed to the influence of endogenous estrogens (1). GlucocorticoidS(GCs) are central to the management of disease activity in SLE; however, their long-term use presents a significant challenge. While they are essential for managing acute flare-ups and organ-threatening situations, prolonged use of GCs is closely associated with a range of adverse effects, most notably Glucocorticoid-induced Osteoporosis(GIOP) (2, 3).Research has demonstrated that prolonged GC therapy in patients with SLE results in substantial declines in bone mineral density and microarchitecture, which are positively correlated with cumulative organ damage (4). Osteoporosis (OP), as one of the most serious complications of SLE, This OP not only increases the risk of fractures but may also contribute to the overall disease burden in individuals with SLE (5). As a significant complication of SLE, epidemiological studies reveal a considerably higher prevalence of OP in SLE patients compared to the general population (2, 3). It is important to note that the etiology of OP in SLE patients is multifactorial and not solely due to GCs use. The chronic inflammatory state of the disease, immune dysregulation, altered sex hormone levels, vitamin D deficiency, renal involvement, and prolonged disease duration have all been identified as significant independent risk factors (6–10). Moreover, GCs not only influence osteoclast function but may also aggravate SLE by disrupting immune system equilibrium (11). Therefore, achieving an optimal balance in GCs administration to minimize adverse effects while effectively managing disease activity remains a significant clinical challenge (12).

Despite extensive research, the exact magnitude of the global burden of OP in SLE and its primary determinants remain inadequately quantified and contentious. This is primarily due to significant heterogeneity in sample sizes, diagnostic criteria, and methodologies across existing studies. A critical and unresolved question persists: does SLE-related bone loss exhibit a site-specific predilection? This question poses a fundamental clinical dilemma—should the lumbar spine, femoral neck, and total hip be equally prioritized during bone mineral density (BMD) monitoring? Addressing this gap is essential for transitioning from a non-specific screening approach to a targeted, risk-stratified management paradigm. To address these evidence gaps, we conducted a comprehensive systematic review and meta-analysis, utilizing what we believe to be the largest aggregated global dataset on this topic. Our objectives were threefold: to definitively establish the overall and site-specific prevalence of OP and osteopenia in SLE; to identify high-risk populations through rigorous subgroup analyses; and to quantify the impact of key risk factors, particularly glucocorticoid exposure, on site-specific bone loss.

## Materials and methods

### Literature search

We conducted a comprehensive systematic search of both Chinese (CNKI, WANFANG Data) and international (PubMed, Embase, Cochrane Library, Web of Science) databases. The search was performed using both subject headings and free-text terms, covering studies published up to September 26, 2025. The review protocol was registered with the International Platform of Registered Systematic Review and Meta-analysis Protocols (INPLASY), ensuring adherence to standardized guidelines for systematic reviews (registration number: INPLASY2025120043; https://doi.org/10.37766/inplasy2025.12.0043). Studies were included based on stringent eligibility criteria, focusing on observational studies that report the prevalence of OP or osteopenia in patients with SLE, as well as the associated risk factors. The detailed search strategy is provided in **Supplementary Material Method 1**.

### Inclusion and exclusion criteria for literature

#### Inclusion criteria

(1) Participants must satisfy the 1997 revised classification criteria for SLE as established by the American College of Rheumatology (ACR); (2) OP must be diagnosed in accordance with the World Health Organization (WHO) criteria, utilizing dual-energy X-ray absorptiometry (DXA), whereby individuals are categorized as: Normal (T-score ≥ -1.0), Osteopenia (-2.5 < T-score < -1.0), or OP (T-score ≤ -2.5); (3) The scope of studies is confined to those published in either Chinese or English; (4) The study designs considered include cross-sectional studies, cohort studies, or case-control studies.

#### Exclusion criteria

(1) A history of utilizing estrogens, androgens, anticoagulants, and medications influencing bone metabolism; (2) Significant hepatic or renal impairment, Cushing’s syndrome, a history of thyroid or parathyroid disorders, or a history of oophorectomy; (3)Animal studies, articles lacking full-text availability, theses, conference proceedings, abstracts, and similar sources.

### Data extraction and quality assessment

Two independent reviewers (YCH and DSY) conducted a systematic screening of the literature. Data extraction was carried out utilizing a standardized form that included details such as the first author, year of publication, diagnostic criteria, study population, study design, sample size, and potential confounding factors. The extracted data were subsequently cross-verified, and any discrepancies were addressed through consultation with the corresponding author (MXL).

### Statistical methods

Statistical analyses of the prevalence and determinants of SLE with OP were conducted using Stata 12.0 software. Heterogeneity among the included studies was assessed, with a P-value of ≥ 0.1 and an I² statistic of < 50% indicating no significant statistical heterogeneity, thereby justifying the use of a fixed-effect model. Conversely, a P-value of < 0.1 and an I² statistic of ≥ 50% suggested significant heterogeneity, warranting the application of a random-effects model for the meta-analysis. Subgroup analyses were performed to identify potential sources of heterogeneity. Sensitivity analyses were conducted using sequential exclusion methods. Publication bias was evaluated through funnel plots and the Egger test. Statistical significance was determined at a P-value of < 0.05.

## Results

**Figure 1:**
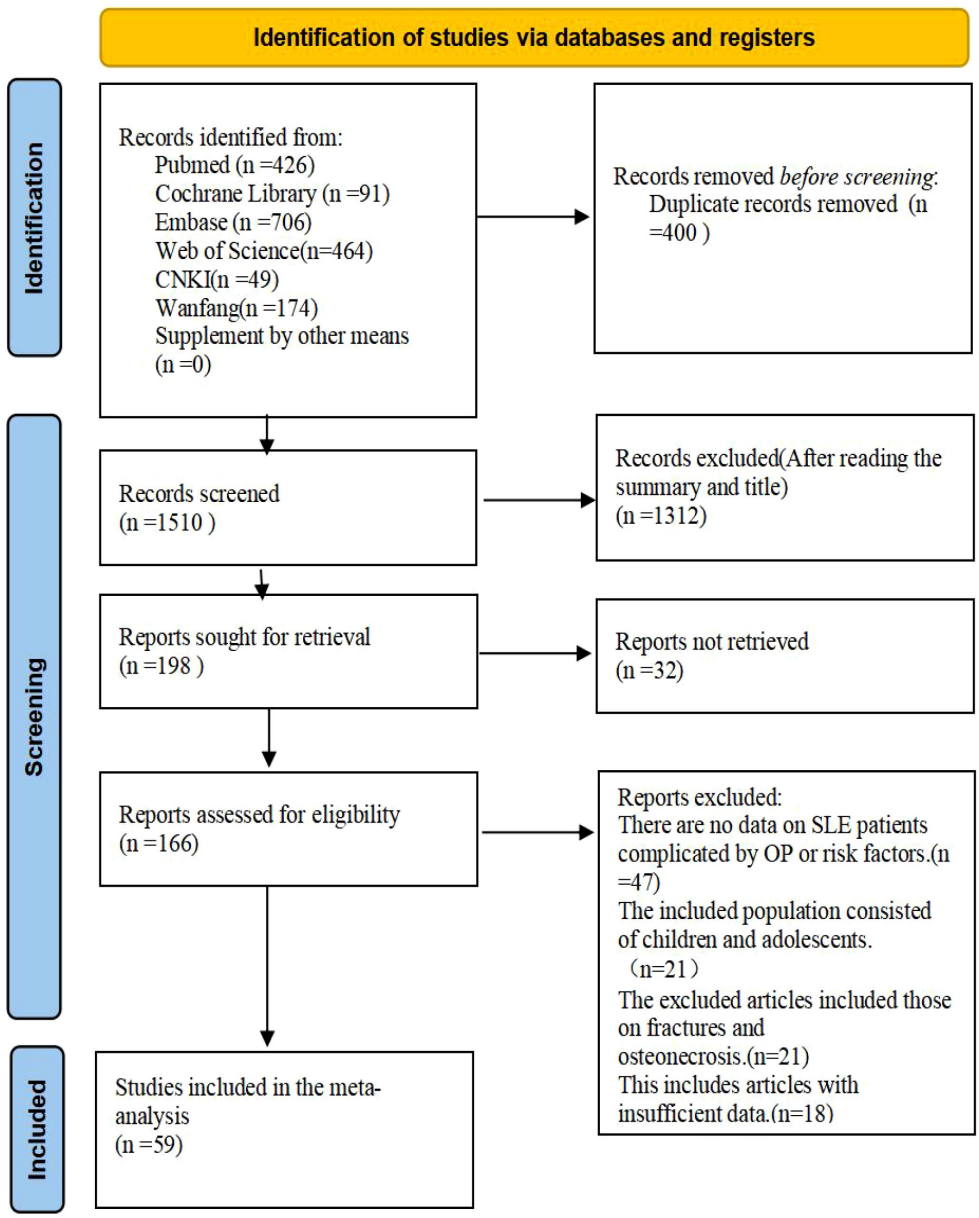
The process of article inclusion.

**Figure 2:**
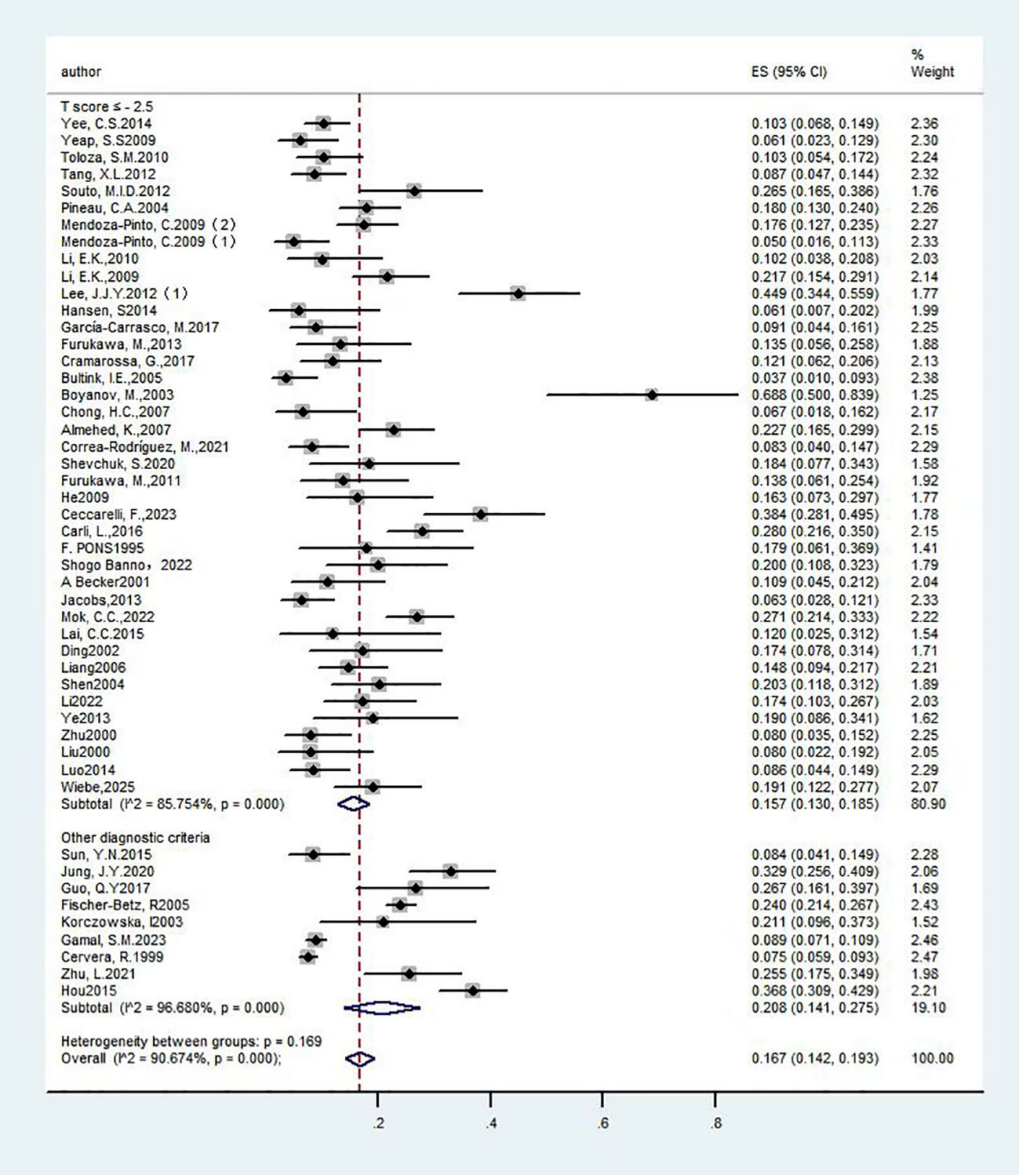
Forest plot: Overall prevalence of osteoporosis in systemic lupus erythematosus. (T score ≤ - 2.5 and Other diagnostic criteria).

Results of the Literature Search The initial search identified a total of 1,910 articles. After the removal of 400 duplicate entries, 1,312 articles were excluded based on the screening of titles and abstracts. Additionally, 32 articles were not available in full-text format. Following a comprehensive review of the full texts, 107 articles were further excluded. Consequently, 59 articles were deemed suitable for inclusion in the analysis. The process of article inclusion is depicted in **Figure 1** and **Figure 2** summary table of all included articles is provided in **Supplementary Tables 1**-**3**.

### Basic characteristics and quality assessment of included studies

This review encompassed a total of 59 articles, comprising 48 cross-sectional studies, 3 case-control studies, and 8 cohort studies. The quality of the studies was independently assessed by two reviewers utilizing the Newcastle-Ottawa Scale (NOS) and the Agency for Healthcare Research and Quality (AHRQ) assessment methods. Specifically, the NOS was applied to evaluate the quality of the case-control and cohort studies. The NOS framework assesses these studies across three domains, consisting of eight items: selection of the study population, comparability, and assessment of exposure or outcome. It employs a semi-quantitative star-rating system to gauge the quality of the literature, with a maximum score of nine stars. Studies achieving a score of 7 or more stars are categorized as ‘high-quality research,’ those with scores of 5 to 6 stars are deemed ‘moderate-quality research,’ and those scoring 4 stars or fewer are considered ‘low-quality research. ‘The quality of literature pertaining to cross-sectional studies was evaluated using the Agency for Healthcare Research and Quality (AHRQ) scale. This scale consists of 11 items, each assessed using a three-tier evaluation system: ‘yes’, ‘no’, or ‘unclear’, with scores directly reflecting the quality of the study. A ‘yes’ response to an item yields 1 point, while ‘no’ or ‘unclear’ responses yield 0 points. The maximum possible score on the AHRQ scale is 11 points, with scores of 8 or higher indicating ‘high-quality research’, scores of 6–7 indicating ‘moderate-quality research’, and scores of 5 or lower indicating ‘low-quality research’. In this assessment, 13 articles were categorized as high-quality, 18 as moderate-quality, and 28 as low-quality. Detailed scoring information is provided in **Supplementary Tables 4**, **5**.

### Meta-analysis results

#### The prevalence of OP among individuals with SLE

A total of forty-nine studies (13–61) investigated the overall prevalence of OP among patients with SLE, encompassing 7,666 participants. The studies exhibited substantial heterogeneity (I²=90.7%), warranting the application of a random-effects model. The analysis revealed a prevalence rate of 16.70% [95% CI (14.2%, 19.3%), P<0.01], indicating a statistically significant difference. Detailed findings are provided in **Figure 2** and **Table 1**.

**Table 1:** Summary of overall prevalence of OP in patients with SLE.

Subgroup name	Number of people with OP	Total sample size(n)	Detection rates/(95%CI)(%)	P Value	I2(%)	Effect model	Egger
Total detection rate	1267	7666	16.7 (14.2, 19.3)	P<0.01	90.7%	random	0.001
Diagnostic Criteria							
T score ≤ - 2.5	656	3992	15.7 (13, 18.5)	P<0.01	85.8%	random	
Other diagnostic criteria	611	3674	20.8 (14.1, 27.5)	P<0.01	96.7%	random	
Sex							
Mixed-gender group	614	4183	14.7 (10.3, 19.2)	P<0.01	94.2%	random	
Female-only group	637	3423	17.4 (14, 20.8)	P<0.01	88.1%	random	
Male-only group	16	60	26.7 (16.1, 39.7)	P<0.01	/	random	
Area							
Europe	564	3407	17.9 (12.9, 23)	P<0.01	94.2%	random	
Asia	453	2367	16.5 (12.7, 20.3)	P<0.01	85.9%	random	
North America	152	922	15.9 (9.1, 22.6)	P<0.01	90.0%	random	
South America	18	68	26.5 (16.5, 38.6)	P<0.01	/	random	
Africa	80	902	8.9 (7, 10.7)	P<0.01	/	random	
Participants							
Adult group	1069	6388	17.6 (14.4, 20.8)	P<0.01	92.7%	random	
Premenopausal group	119	967	11.6 (8.1, 15)	P<0.01	67.7%	random	
Postmenopausal females plus males aged > 50 years	32	184	16.6 (7.3, 26)	P<0.01	63.6%	random	
Postmenopausal female	47	127	34.0 (26.1, 41.9)	0.016	90.4%	random	

Based on the overarching prevalence framework, we performed subgroup analyses utilizing various diagnostic criteria to evaluate their influence on prevalence rates. Specifically, employing the diagnostic criterion of a T-score ≤ -2.5, 40 studies (13–16, 18–24, 26, 28, 29, 31–37, 40, 41, 43–47, 49–58, 60, 61) reported on the prevalence of OP among individuals with SLE, encompassing a total of 3,992 participants. Considerable heterogeneity was observed among these studies (I²=85.8%), which warranted the application of a random-effects model. The findings revealed a prevalence rate of 15.70% [95% CI (13%, 18.5%), P<0.01], indicating a statistically significant difference. Separately, nine articles (17, 25, 27, 30, 38, 39, 42, 48, 59) reported the prevalence of OP in patients with SLE under other diagnostic criteria, involving 3,674 subjects. Heterogeneity was substantial across studies (I²=96.7%), necessitating a random-effects model. Results indicated a prevalence of 20.80% [95% CI (14.1%, 27.5%), P<0.01], with statistically significant differences. Findings are presented in **Figure 2** and **Table 1**. Thus, variations in diagnostic criteria exert a discernible influence on prevalence.

**Figure 3:**
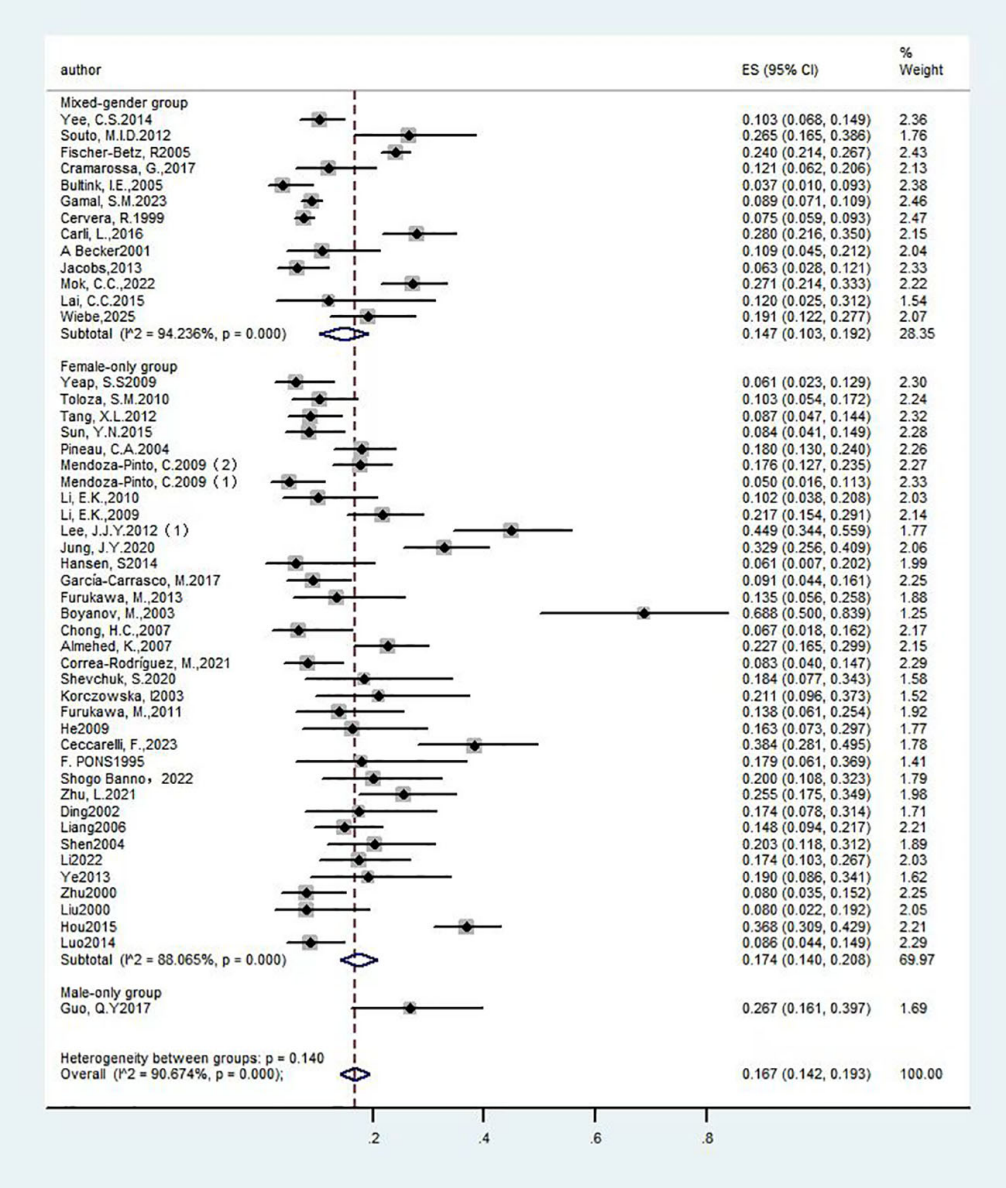
Forest plot: Prevalence of osteoporosis in systemic lupus erythematosus, stratified by sex.

We performed a subgroup analysis based on patient gender across the included studies to assess differences in prevalence between genders. Thirteen studies (13, 18, 30–32, 39, 42, 44, 47, 49–51, 61) reported the prevalence of SLE with OP in mixed-gender cohorts, encompassing a total of 4,183 participants. The studies exhibited significant heterogeneity (I²=94.2%), which warranted the use of a random-effects model. The analysis revealed a prevalence rate of 14.70% [95% CI (10.3%, 19.2%), P<0.01], indicating a statistically significant difference. Furthermore, thirty-five studies (14–17, 19–26, 28, 29, 33–38, 40, 41, 43, 45, 46, 48, 52–60) focused on the prevalence of OP in female patients with SLE, involving 3,423 subjects. These studies also demonstrated substantial heterogeneity (I² =88.1%), necessitating a random-effects model. The findings indicated a prevalence rate of 17.40% [95% CI (14.0%, 20.8%), P<0.01], which was statistically significant. These results confirm that women represent a high-risk group for SLE with OP. The detailed results are presented in **Figure 3** and **Table 1**.

**Figure 4:**
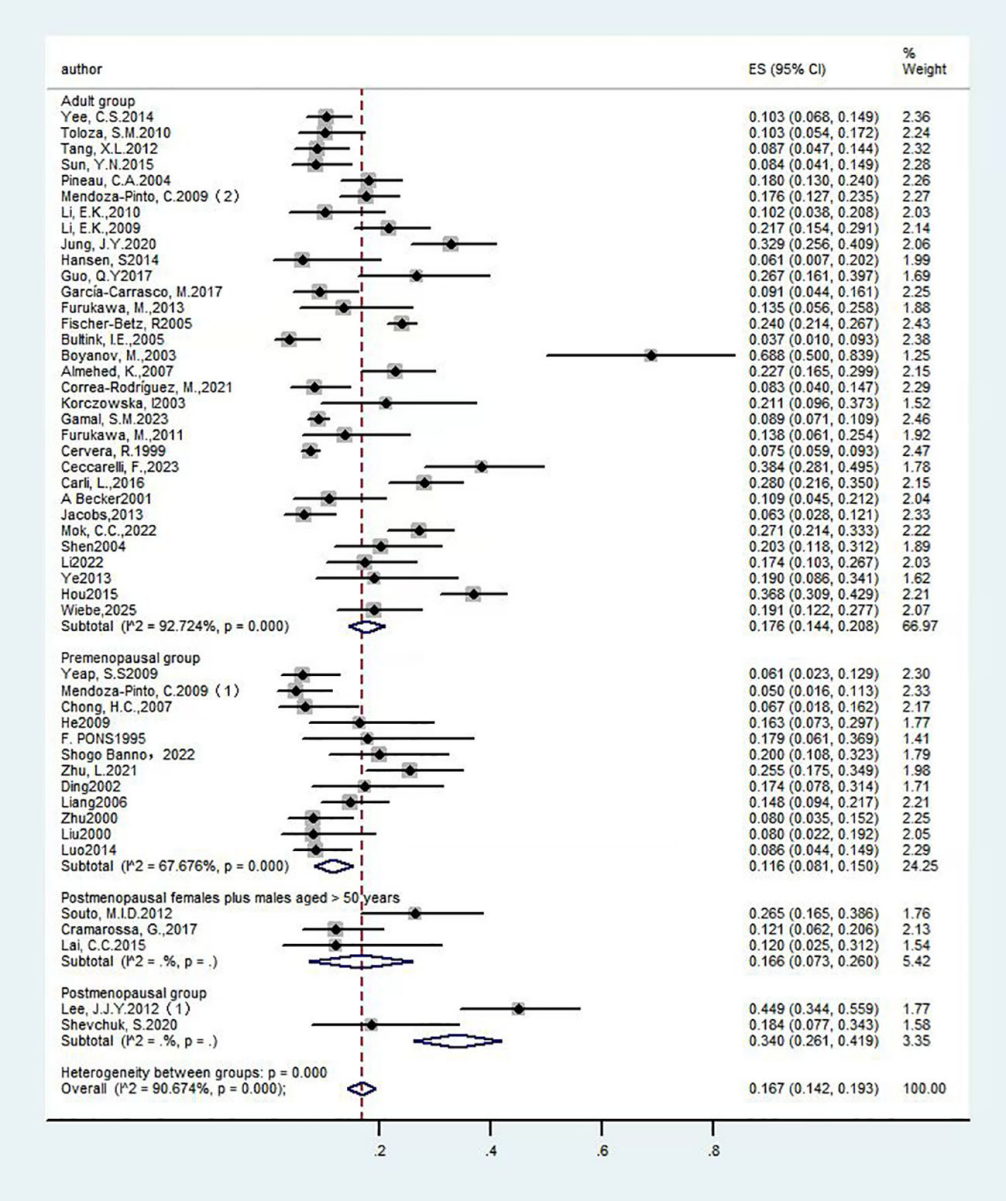
Forest plot: Prevalence of osteoporosis in systemic lupus erythematosus, by patient population.

Among the studies included in the analysis, twelve articles (14, 21, 34, 41, 45, 46, 48, 52, 53, 57, 58, 60) examined the prevalence of OP in premenopausal women diagnosed with SLE, encompassing a total of 967 participants. The studies exhibited significant heterogeneity (I²=67.7%), warranting the application of a random-effects model. The findings revealed a prevalence rate of 11.60% [95% CI (8.1%, 15.0%), P<0.01], indicating a statistically significant difference. Additionally, two articles (24, 37) investigated the prevalence of SLE with OP in postmenopausal women, involving 127 participants. The heterogeneity across these studies was substantial (I²=90.4%), also necessitating a random-effects model. The results demonstrated a prevalence rate of 34.0% [95% CI (26.1%, 41.9%),p=0.016], which was statistically significant. Furthermore, three articles (18, 31, 51) reported on the prevalence of SLE with OP in postmenopausal women and men over the age of 50, involving 184 participants. The analysis revealed substantial heterogeneity among the studies (I²=63.6%), warranting the application of a random-effects model. The findings demonstrated a prevalence rate of 16.60% [95% CI (7.3%, 26%), P<0.01], indicating a statistically significant difference. A total of thirty-two studies (13, 15–17, 19, 20, 22, 23, 25–30, 32, 33, 35, 36, 38–40, 42–44, 47, 49, 50, 54–56, 59, 61) provided data on the prevalence of OP in adults with SLE across various age groups, encompassing 6,388 participants. The studies exhibited significant heterogeneity (I²=92.7%), necessitating the use of a random-effects model. The results indicated a prevalence rate of 17.6% [95% CI (14.4%, 20.8%), P<0.01], which was statistically significant. In conclusion, postmenopausal women show a further increased prevalence of SLE with OP compared to other populations. The detailed results are provided in **Figure 4** and **Table 1**.

**Figure 5:**
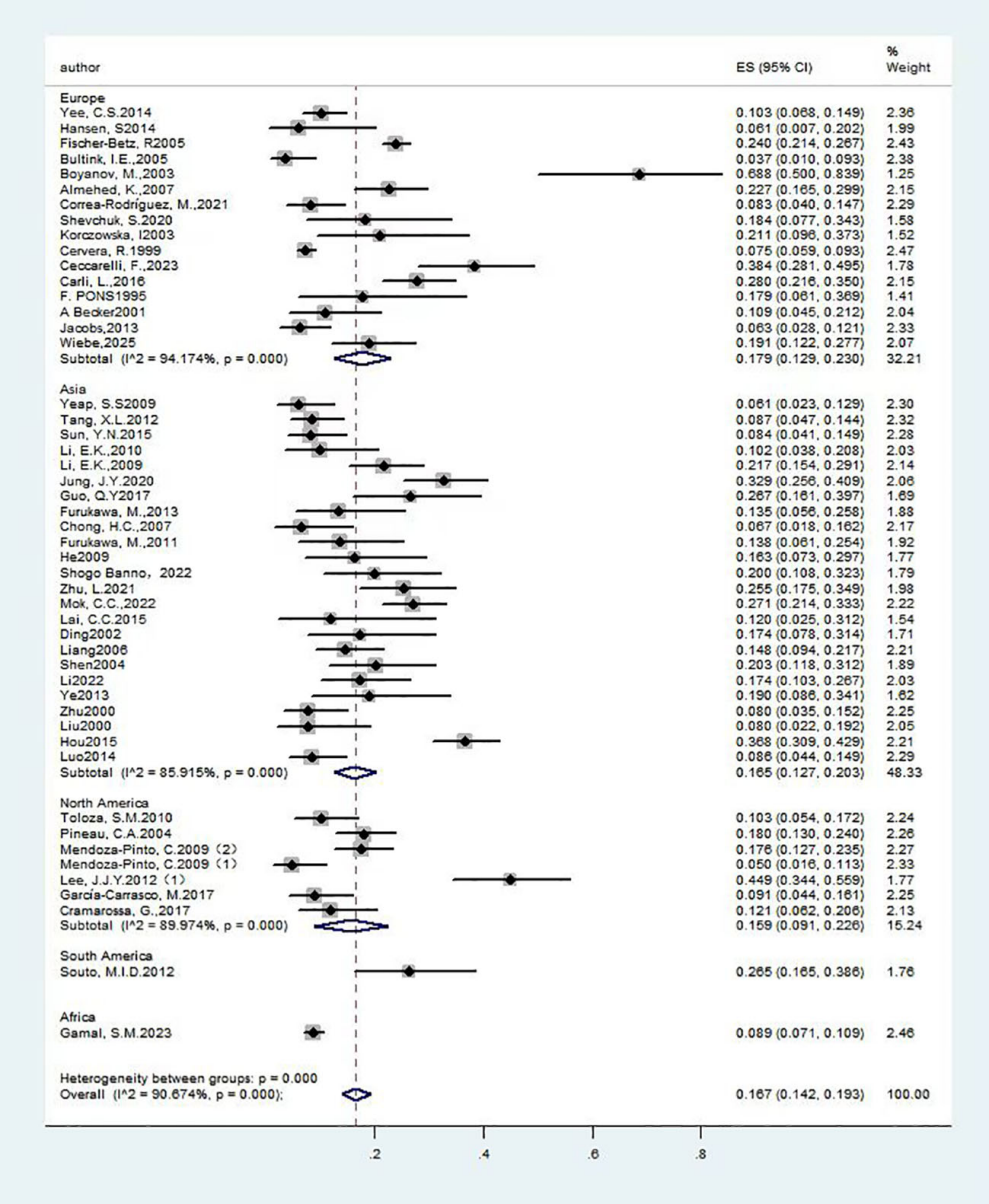
Forest plot: Prevalence of osteoporosis in systemic lupus erythematosus, by study region.

We performed a subgroup analysis based on the geographical location of the included studies. Sixteen studies (13, 26, 30, 32, 33, 35–38, 42–45, 47, 49, 61) reported the prevalence of OP among individuals with SLE in Europe, encompassing a total of 3,407 subjects. The studies exhibited substantial heterogeneity (I²=94.2%), which warranted the use of a random-effects model. The analysis revealed a prevalence rate of 17.90% [95% CI (12.9%, 23.0%), P<0.01], indicating a statistically significant difference. In Asia, twenty-four studies (14, 16, 17, 22, 23, 25, 27, 29, 34, 40, 41, 46, 48, 50–60) assessed the prevalence of OP in SLE patients, involving 2,367 subjects. These studies also demonstrated substantial heterogeneity (I²=85.9%), necessitating a random-effects model. The findings indicated a prevalence rate of 16.50% [95% CI (12.7%, 20.3%), P<0.01], which was statistically significant. Additionally, seven studies (15, 19–21, 24, 28, 31) reported on the prevalence of OP in SLE patients in North America, involving 922 subjects. The analysis revealed substantial heterogeneity among the studies (I²=90.0%), thereby justifying the use of a random-effects model. The findings demonstrated a prevalence rate of 15.90% [95% CI (9.1%, 22.6%), P<0.01], indicating a statistically significant difference. In general, no significant variations in the prevalence of SLE with OP were identified across Eurasia, whereas the Americas exhibited a slightly lower prevalence compared to the overall average. Nevertheless, due to the limited sample size and the number of studies included, these conclusions should be interpreted with caution. Larger-scale studies are necessary for further validation. The detailed results are provided in **Figure 5** and **Table 1**.

### Further investigation into the high heterogeneity of the ‘other diagnostic criteria’ subgroup

A meta-regression analysis was conducted to investigate the high heterogeneity (I² = 96.7%) observed in studies using ‘other diagnostic criteria’ (i.e. those that did not uniformly adopt a T-score ≤ −2.5 SD, but instead used a combination of T-scores, Z-scores or other criteria). The analysis aimed to assess the extent to which four potential covariates (geographical region of the study, gender composition of participants, characteristics of the target population, and sample size) explained this heterogeneity.

The results showed that, of the four variables included, the geographical region in which the studies were conducted was the only statistically significant variable (p = 0.002), indicating that geographical differences are a key factor contributing to the substantial variation in prevalence estimates within this subgroup. In contrast, participant gender composition (p = 0.396), target population classification (p = 0.814) and study sample size (p = 0.507) all showed no significant association.

The significance of the ‘geographical region’ variable strongly suggests that, although studies in different regions were all classified as using ‘other diagnostic criteria’, there may be under-reported differences in the application of specific cut-off values, the selection of population reference databases, or the specific context in which Z-scores were used (e.g. for premenopausal women or young men). These region-specific methodological inconsistencies likely constitute the primary source of heterogeneity in the results within this subgroup. This finding underscores the critical importance, in future studies, of detailing the specific criteria, cut-off values, and reference populations used for the diagnosis of OP, in order to enhance the comparability of results across different studies. The detailed results are provided in **Supplementary Figures 1**-**4**.

### Sensitivity analysis of the overall prevalence of OP in patients with SLE

Due to the considerable heterogeneity observed in our research (I²= 90.7%), we conducted a sensitivity analysis to further explore this issue. The findings revealed a significant decrease in heterogeneity when the study by Boyano et al. (33), which included only 33 patients and therefore had a limited sample size, was excluded. This suggests that sample size may be a contributing factor to the heterogeneity observed in this outcome measure.

A funnel plot was utilized to evaluate publication bias in the study. Specifically, a funnel plot was generated to examine the overall prevalence of SLE in conjunction with OP. The analysis indicated asymmetry on both sides of the funnel plot, supported by an Egger’s test value of 0.001, thereby suggesting the presence of publication bias within the included studies. The detailed results are provided in **Supplementary Figure 5**.

### Prevalence of OP in SLE by anatomical site and subgroup analysis

Whilst conducting a meta-analysis of overall prevalence, we noted that the included studies had performed stratified analyses based on the anatomical site of OP; consequently, we conducted our analysis according to the site of OP.

However, this analysis included only those studies that reported bone mineral density data for the lumbar spine, femoral neck and total hip in the same study, within the same patient cohort. This criterion ensured that comparisons across all sites were conducted within the same study population, thereby controlling for inter-individual and inter-study heterogeneity.

### Prevalence of OP in the lumbar spine

Regarding the lumbar spine, five articles (24, 61–64) reported findings on the prevalence of lumbar OP in patients with systemic lupus erythematosus, involving a total of 463 subjects. There was considerable heterogeneity across the studies (I² = 70.7%), and a random-effects model was employed. The results showed a prevalence of 10.0% [95% CI (5.2%, 14.9%), P < 0.01], with a statistically significant difference; see **Supplementary Figure 6** and **Supplementary Table 6** for the results.

We conducted a subgroup analysis based on the regions where the included studies were conducted. Among these, three articles (61, 63, 64) examined the prevalence of lumbar OP in patients with SLE in Europe; the total number of subjects was 249, with considerable heterogeneity across studies (I² = 73.0%), and a random-effects model was adopted. The results showed a prevalence of 8.10% [95% CI (1.8%, 14.3%), P = 0.012], with a statistically significant difference; one study (62) examined the prevalence of lumbar OP in patients with SLE in Asia, involving a total of 125 subjects. The results showed a prevalence of 15.20% [95% CI (9.4%, 22.7%), P < 0.01]; one study (24) investigated the prevalence of lumbar OP in patients with SLE in North America, involving a total of 89 subjects. The results showed a prevalence of 11.20% [95% CI (5.5%, 19.7%), P < 0.01]; see **Supplementary Figure 6** and **Supplementary Table 6** for the results.

### Prevalence of OP in the femoral neck

Secondly, regarding OP of the femoral neck, five articles (24, 61–64) reported the prevalence of SLE complicated by OP of the femoral neck, involving a total of 463 subjects. Heterogeneity among the studies was low (I² = 0%), and a fixed-effects model was adopted. The results showed a prevalence of 7.0% [95% CI (4.6%, 9.3%), P < 0.01], with a statistically significant difference; see **Supplementary Figure 7** and **Supplementary Table 7** for the results.

We conducted a subgroup analysis based on the regions where the included studies were conducted. Among these, three articles (61, 63, 64) investigated the prevalence of SLE complicated by osteoporotic femoral neck fractures in Europe, involving a total of 249 subjects. Heterogeneity among the studies was low (I² = 0%), and a fixed-effects model was adopted. The results showed a prevalence of 7.00% [95% CI (3.9%, 10.2%), P < 0.01], with a statistically significant difference; one study (62) investigated the prevalence of SLE complicated by osteoporotic femoral neck fractures in Asia, involving a total of 125 subjects. The results showed a prevalence of 8.10% [95% CI (3.3%, 13.0%), P < 0.01]; one article (24) investigated the prevalence of SLE complicated by osteoporotic femoral neck fractures in North America, with a total of 89 subjects. The results showed a prevalence of 5.60% [95% CI (0.8%, 10.4%), P < 0.01]; see **Supplementary Figure 7** and **Supplementary Table 7** for the results.

### Prevalence of OP in the total hip

Regarding the total hip, five articles (24, 61–64) reported findings on the prevalence of total hip OP in patients with systemic lupus erythematosus, involving a total of 463 subjects. Heterogeneity among the studies was low (I² = 0%), and a fixed-effects model was employed. The results showed a prevalence of 5.30% [95% CI (3.3%, 7.3%), P < 0.01], with a statistically significant difference; see **Supplementary Figure 8** and **Supplementary Table 8** for the results.

We conducted a subgroup analysis based on the regions where the included studies were conducted. Among these, three articles (61, 63, 64) investigated the prevalence of SLE complicated by total hip OP in Europe, involving a total of 453 subjects, with low heterogeneity across the studies (I² = 0%). The results showed a prevalence of 5.40% [95% CI (2.6%, 8.2%), P < 0.01], with a statistically significant difference; one study (62) investigated the prevalence of SLE complicated by total hip OP in Asia, involving a total of 125 subjects. The results showed a prevalence of 8.20% [95% CI (4.0%, 14.6%), P < 0.01]; one study (24) investigated the prevalence of SLE complicated by total hip OP in North America, with a total of 89 subjects. The results showed a prevalence of 3.40% [95% CI (0.7%, 9.5%), P = 0.078]; see **Supplementary Figure 8** and **Supplementary Table 8** for the results.

Overall, within the scope of the data included in this study, the lumbar spine is the site with the highest risk of OP in SLE patients. In terms of regional distribution, point estimates of prevalence for all sites indicate that the Asian region is higher than Europe and North America. However, it must be clearly noted that the subgroup analyses for Asia and North America were based on data from a single study each, whereas the European subgroup was based on three studies. This significant imbalance in sample size, coupled with potential population-specific biases in individual studies, means that the differences in point estimates observed between regions cannot yet be considered definitive epidemiological evidence. These findings are merely exploratory, suggesting that there may be regional variations in the prevalence of SLE-associated OP; however, the exact patterns, magnitude and causes require confirmation by future large-scale, multicenter, standardized studies. When interpreting these results, priority should be given to the pooled estimates from the European subgroup (based on multiple studies), whilst the results from the single studies in Asia and North America should be treated with caution.

### Sensitivity analysis and publication bias regarding the prevalence of OP in different sites in patients with SLE

Given the substantial heterogeneity in our study, we conducted a sensitivity analysis. Heterogeneity regarding the prevalence of lumbar OP was (I² = 70.7%), The results of the sensitivity analysis showed that heterogeneity decreased significantly after excluding the study by Salman-Monte et al., 2015 (63), as this study included only 66 patients and had a small sample size; we therefore speculate that sample size may be a source of heterogeneity for this outcome measure. The results are shown in **Supplementary Figure 9**.

A funnel plot was used to assess publication bias. A funnel plot was constructed for the prevalence of SLE complicated by OP, stratified by anatomical site. The results showed asymmetry on both sides of the funnel plot, with an Egger’s test p-value of 0.13 for the lumbar spine, 0.617 for the femoral neck, and 0.298 for the total hip. Consequently, publication bias in the included literature was not significant; the results are shown in **Supplementary Figures 10**-**12**.

### The prevalence of Osteopenia among individuals with SLE

**Figure 6:**
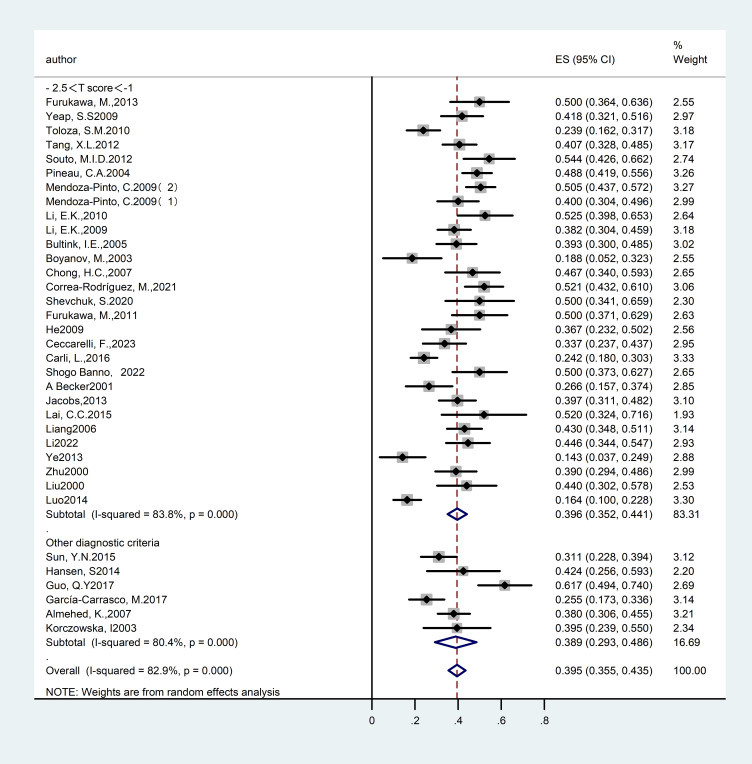
Forest plot: Overall prevalence of osteopenia in systemic lupus erythematosus. (-2.5<T score <- 1 and Other diagnostic criteria).

Reduction in bone mass serves as an early indicator of OP, with individuals experiencing this condition potentially advancing to full OP over time. A review of existing literature reveals numerous studies documenting patients who do not yet fulfill the diagnostic criteria for OP but are instead in the initial phase of bone mass reduction. In response, we conducted an analysis of the prevalence of SLE in conjunction with bone mass reduction to thoroughly assess the impact of SLE on patients’ bone mineral density. A total of thirty-five studies (14–23, 26–29, 32–38, 40, 41, 43, 44, 46, 47, 49, 51, 53, 55–58, 60) were identified, encompassing 3,300 subjects and reporting on the overall prevalence of reduced bone mass in individuals with SLE. Due to significant heterogeneity among the studies (I²= 82.9%), a random-effects model was employed for the analysis. The results demonstrated a prevalence rate of 39.50% [95% CI (35.5%, 43.5%), P<0.01], indicating a statistically significant difference. The detailed results are provided in **Figure 6** and **Table 2**.

**Table 2:** Summary of overall prevalence of osteopenia in patients with SLE.

Subgroup name	Number of individuals with reduced bone mass	Total sample size(n)	Detection rates/(95%CI)(%)	P Value	I2(%)	Effect model	Egger
Total detection rate	1300	3300	39.5(35.5, 43.5)	P<0.01	82.9%	random	0.043
Diagnostic Criteria							
- 2.5<T score<-1	1107	2777	39.6(35.2, 44.1)	P<0.01	83.8%	random	
Other diagnostic criteria	193	523	38.9(29.3, 48.6)	P<0.01	80.4%	random	
Sex							
Mixed-gender group	204	576	38.2(28.4, 47.9)	P<0.01	82.9%	random	
Female-only group	1059	2664	39.0(34.7, 43.4)	P<0.01	82.3%	random	
Male-only group	37	60	61.7(49.4, 74)	P<0.01	/	random	
Participants							
Adult group	931	2382	38.3(33.5, 43.1)	P<0.01	83.8%	random	
Premenopausal group	300	787	39.3(30.9, 47.6)	P<0.01	83.9%	random	
Postmenopausal females plus males aged > 50 years	50	93	53.8(43.6, 63.9)	P<0.01	0%	random	
Postmenopausal female	19	38	50(34.1, 65.9)	P<0.01	/	random	
Area							
Europe	362	994	36.4(30.2, 42.6)	P<0.01	75.6%	random	
Asia	599	1496	41.2(35.1, 47.2)	P<0.01	83.5%	random	
North America	302	742	37.8(26.4, 49.3)	P<0.01	90.9%	random	
South America	37	68	54.4(42.6, 66.2)	P<0.01	/	random	

In light of the overall prevalence, we performed subgroup analyses utilizing various diagnostic criteria to evaluate their influence on prevalence rates. A total of twenty-nine studies (14–16, 18–23, 29, 32–34, 36, 37, 40, 41, 43, 44, 46, 47, 49, 51, 53, 55–58, 60) reported on the prevalence of SLE with associated reduced bone mass, encompassing 2,777 participants. The studies exhibited considerable heterogeneity (I²=83.8%), warranting the application of a random-effects model. The analysis revealed a prevalence rate of 39.60% [95% CI (35.2%, 44.1%), P<0.01], indicating a statistically significant difference. Under alternative diagnostic criteria, six studies (17, 26–28, 35, 38) examined the prevalence of SLE associated with reduced bone mass, encompassing a total of 523 subjects. The heterogeneity among these studies was also substantial (I²= 80.4%), necessitating the use of a random-effects model. The findings indicated a prevalence rate of 38.9% (95% CI: 29.3%, 48.6%; P<0.01), demonstrating statistically significant differences. Consequently, the variation in diagnostic criteria did not markedly affect the overall prevalence, suggesting the robustness of the results. The detailed results are provided in **Figure 6** and **Table 2**.

**Figure 7:**
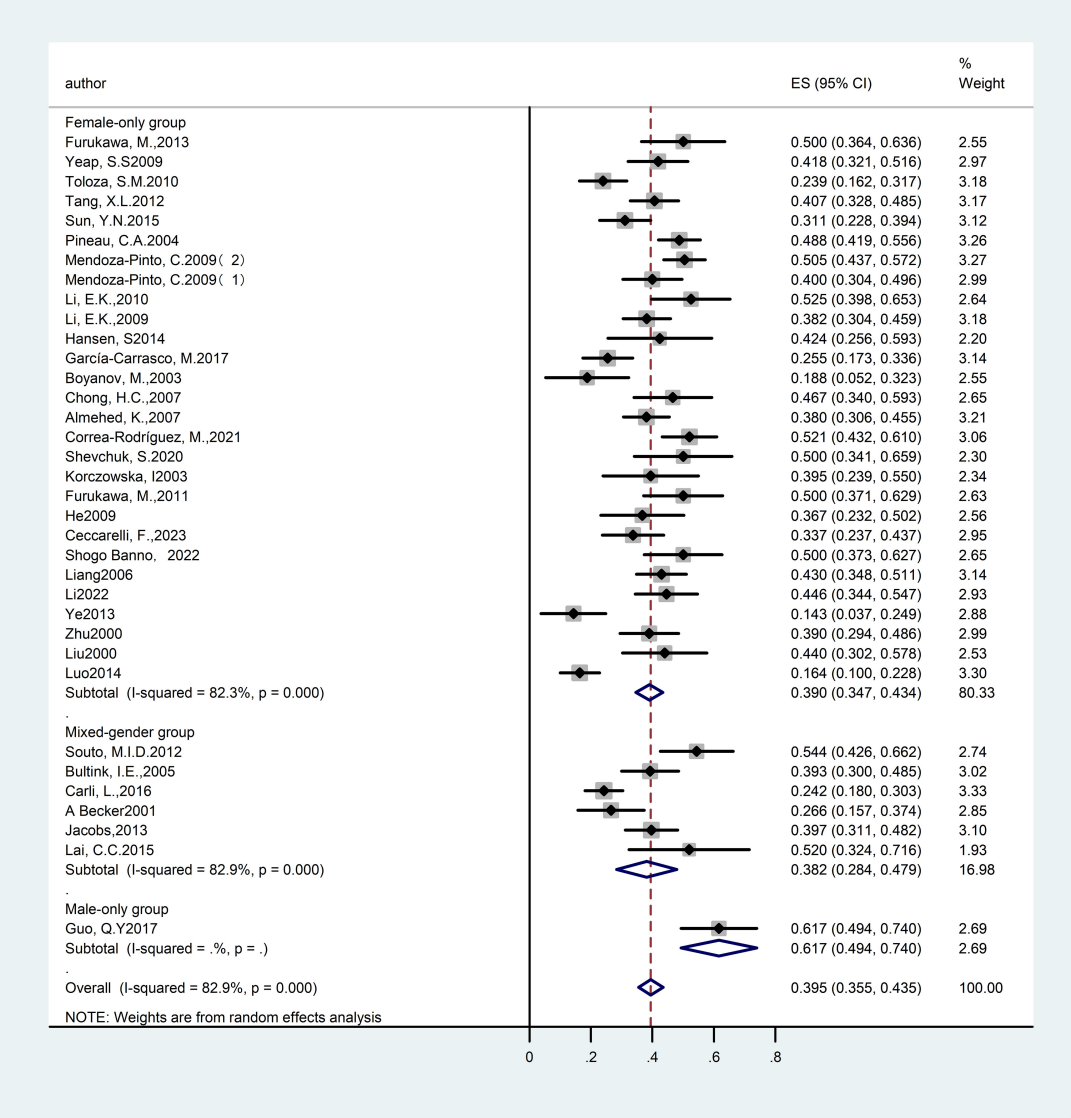
Forest plot: Prevalence of osteopenia in systemic lupus erythematosus, stratified by sex.

A subgroup analysis was performed to examine gender differences in prevalence across the included studies. Six studies (18, 32, 44, 47, 49, 51) provided data on the prevalence of SLE associated with reduced bone mass in mixed-gender cohorts, encompassing a total of 576 participants. The analysis revealed substantial heterogeneity among the studies (I²= 82.9%), which warranted the application of a random-effects model. The results demonstrated a prevalence rate of 38.20% [95% CI (28.4%, 47.9%), P<0.01], indicating a statistically significant difference. A total of twenty-eight studies (14–17, 19–23, 26, 28, 29, 33–38, 40, 41, 43, 46, 53, 55–58, 60) examined the prevalence of SLE associated with reduced bone mass in women, encompassing 2,664 participants. The analysis revealed substantial heterogeneity among the studies (I²= 82.3%), which was addressed using a random-effects model. The prevalence was found to be 39.00% [95% CI (34.7%, 43.4%), P<0.01], signifying a statistically significant difference. Due to the lack of explicit differentiation between male and female participants in the included studies for this outcome measure, a comparison was conducted across the entire population. The detailed results are provided in **Figure 7** and **Table 2**.

**Figure 8:**
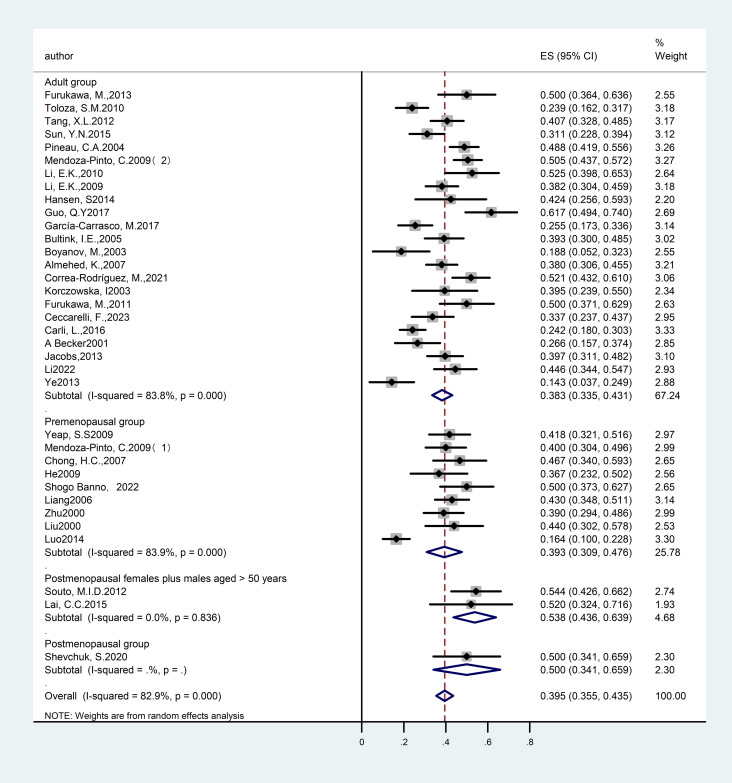
Forest plot: Prevalence of osteopenia in systemic lupus erythematosus, by patient population.

Among the studies included in the analysis, nine articles (14, 21, 34, 41, 46, 53, 57, 58, 60) examined the prevalence of SLE associated with reduced bone mass in premenopausal women, encompassing a total of 887 participants. The studies exhibited significant heterogeneity (I²=83.9%), which warranted the application of a random-effects model. The findings revealed a prevalence rate of 39.30% [95% CI 30.9%, 47.6%, P<0.01], indicating a statistically significant difference. Additionally, two articles (18, 51) investigated the prevalence of SLE with reduced bone mass in postmenopausal women and men over 50 years of age, involving 93 participants. The studies exhibited considerable heterogeneity (I²=0%). The findings revealed a prevalence rate of 53.80% [95% CI (43.6%, 63.9%), P<0.01], indicating a statistically significant difference. In conclusion, our analysis suggests that postmenopausal women and men over the age of 50 constitute high-risk groups for SLE associated with reduced bone mass, with a prevalence rate surpassing the average observed in other age cohorts. The detailed results are provided in **Figure 8** and **Table 2**.

**Figure 9:**
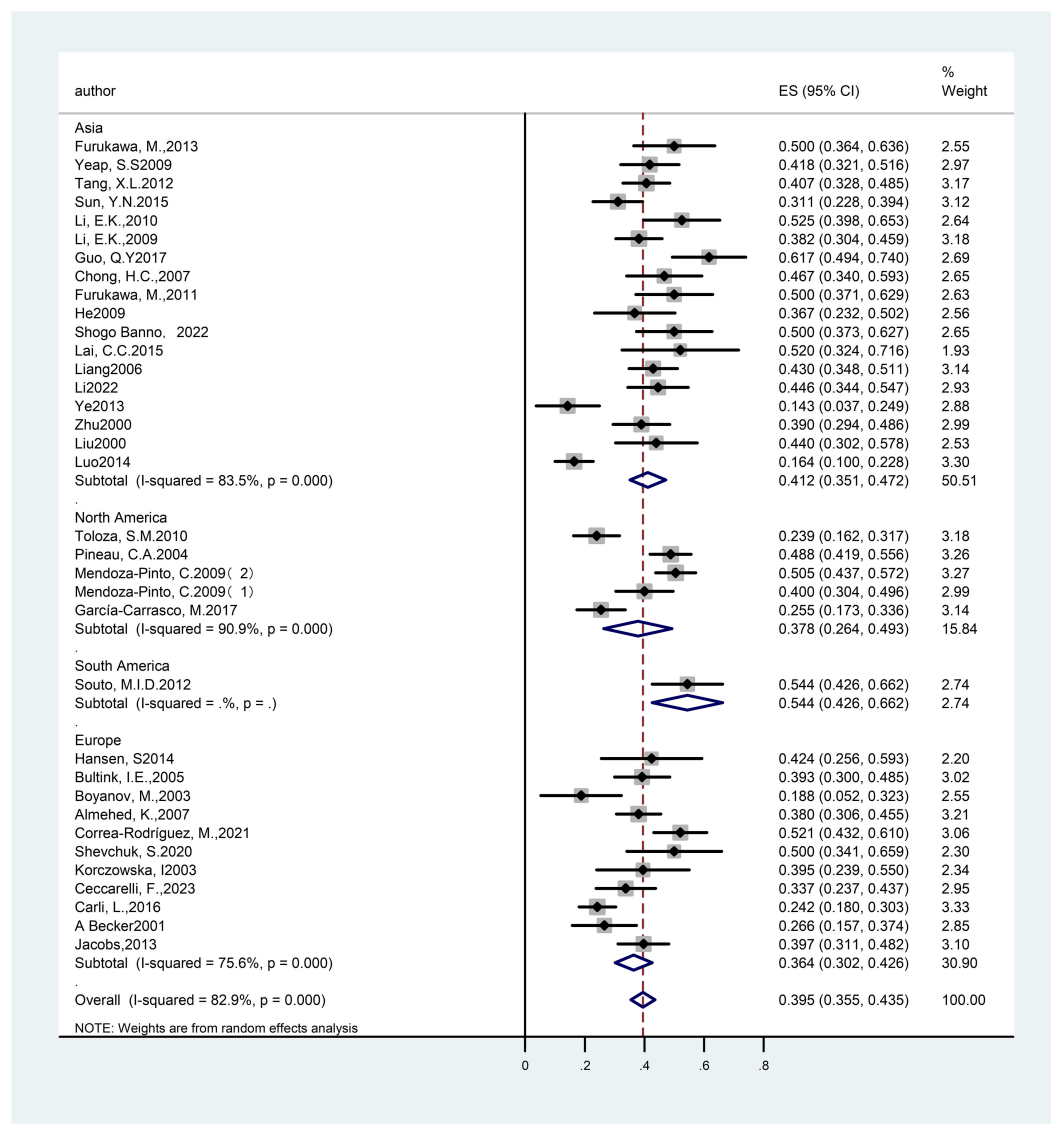
Forest plot: Prevalence of osteopenia in systemic lupus erythematosus, by study region.

We performed a subgroup analysis stratified by the geographical location of the studies included in our review. Eleven studies (26, 32, 33, 35–38, 43, 44, 47, 49), encompassing a total of 994 participants, provided data on the prevalence of SLE associated with bone loss in European populations. The heterogeneity among these studies was considerable (I²= 75.6%), which warranted the application of a random-effects model. The analysis revealed a prevalence rate of 36.40% [95% CI (30.2%, 42.6%), P<0.01)]. Additionally, eighteen studies (14, 16, 17, 22, 23, 27, 29, 34, 40, 41, 46, 51, 53, 55–58, 60) reported on the prevalence of SLE with reduced bone mass in Asian populations, involving a total of 1,496 subjects. The studies exhibited considerable heterogeneity (I²=83.5%), necessitating the application of a random-effects model. The findings revealed a prevalence rate of 41.20% [95% CI (35.1%, 47.2%), P<0.01], indicating a statistically significant difference. Five studies (15, 19–21, 28) provided data on the prevalence of SLE associated with reduced bone mass in North America, encompassing a total of 742 participants. The heterogeneity across these studies was substantial (I²= 90.9%), prompting the use of a random-effects model. The results demonstrated a prevalence of 37.80% [95% CI (26.4%, 49.3%), P<0.01], which was statistically significant. Overall, the prevalence of SLE with reduced bone mass was found to be higher in Asia compared to North America and Europe, indicating that geographical factors should be taken into account when interpreting these results. This finding aligns with previous research on OP, the detailed results are provided in **Figure 9** and **Table 2**.

### Sensitivity analysis and publication bias regarding the overall prevalence of reduced bone mass in SLE

Given the considerable heterogeneity observed in our study, we conducted a sensitivity analysis; the heterogeneity regarding overall prevalence was (I² = 82%). The results of the sensitivity analysis showed that heterogeneity decreased significantly after excluding the study by Guo et al. (27), as this study included only 60 patients and had a small sample size; we therefore speculate that sample size may be a source of heterogeneity for this outcome measure. The results are provided in **Supplementary Figure 13**.

A funnel plot was employed to assess publication bias. A funnel plot was constructed for the overall prevalence of systemic lupus erythematosus with reduced bone mass. The results showed asymmetry on both sides of the funnel plot, with an Egger’s test p-value of 0.038; consequently, there is some publication bias among the included studies. The results are shown in **Supplementary Figure 14**.

### Prevalence of osteopenia in systemic lupus erythematosus by anatomical site and subgroup analysis

In addition to conducting a meta-analysis of the overall prevalence, we performed a stratified analysis of the sites of osteopenia reported in the included studies. Consistent with the site-specific analysis for OP, this analysis included only those studies that reported bone mineral density data for the lumbar spine, femoral neck and total hip simultaneously within the same study and for the same patient cohort. This criterion ensured that comparisons across all sites were conducted within the same study population, thereby controlling for inter-individual and inter-study heterogeneity.

### Prevalence of osteopenia in the lumbar spine

Regarding the lumbar spine, five articles (24, 61–64) reported findings on the prevalence of lumbar bone loss in patients with systemic lupus erythematosus, involving a total of 463 subjects. Heterogeneity across the studies was low (I² = 0%), and a fixed-effects model was employed. The results showed a prevalence of 35.60% [95% CI (31.3%, 40.0%), P < 0.01], with a statistically significant difference; see **Supplementary Figure 15** and **Supplementary Table 9** for the results.

We conducted a subgroup analysis based on the regions where the included studies were conducted. Among these, three articles (61, 63, 64) investigated the prevalence of lumbar bone loss in patients with systemic lupus erythematosus in Europe, involving a total of 249 subjects, with low heterogeneity across the studies (I² = 21.4%). The results showed a prevalence of 34.60% [95% CI (28.7%, 40.5%), P < 0.01], with a statistically significant difference; one study (62) investigated the prevalence of lumbar bone loss in patients with systemic lupus erythematosus in Asia, involving a total of 125 subjects. The results showed a prevalence of 38.40% [95% CI (29.9%, 46.9%), P < 0.01]; one article (24) investigated the prevalence of lumbar bone loss in patients with systemic lupus erythematosus in North America, with a total of 89 subjects. The results showed a prevalence of 34.80% [95% CI (24.9%, 44.7%), P < 0.01]; see **Supplementary Figure 15** and **Supplementary Table 9** for the results.

### Prevalence of osteopenia in the femoral neck

Regarding studies on SLE with femoral neck bone loss, five articles (24, 61–64) reported the prevalence of femoral neck bone loss in patients with systemic lupus erythematosus, involving a total of 463 subjects. Heterogeneity among the studies was low (I² = 0%), and a fixed-effects model was adopted. The results showed a prevalence of 44.10% [95% CI (39.6%, 48.6%), P < 0.01], with a statistically significant difference; see **Supplementary Figure 16** and **Supplementary Table 10** for the results.

We conducted a subgroup analysis based on the regions where the included studies were conducted. Among these, three articles (61, 63, 64) investigated the prevalence of femoral neck bone loss in patients with systemic lupus erythematosus in Europe, involving a total of 249 subjects, with low heterogeneity across the studies (I² = 37.7%). The results showed a prevalence of 42.80% [95% CI (36.7%, 48.9%), P < 0.01], with a statistically significant difference; one study (62) investigated the prevalence of femoral neck bone loss in patients with systemic lupus erythematosus in Asia, involving a total of 125 subjects. The results showed a prevalence of 46.30% [95% CI (37.5%, 55.2%), P < 0.01]; one study (24) investigated the prevalence of femoral neck bone loss in patients with systemic lupus erythematosus in North America, involving a total of 89 subjects. The results showed a prevalence of 44.90% [95% CI (34.6%, 55.3%), P < 0.01]; see **Supplementary Figure 16** and **Supplementary Table 10** for the results.

### Prevalence of osteopenia in the total hip

Regarding studies on SLE combined with total hip bone loss, five articles (24, 61–64) reported the prevalence of total hip bone loss in patients with systemic lupus erythematosus, involving a total of 463 subjects. Heterogeneity among the studies was low (I² = 21.8%), and a fixed-effects model was employed. The results showed a prevalence of 36.70% [95% CI (32.3%, 41.1%), P < 0.01], with a statistically significant difference; see **Supplementary Figure 17** and **Supplementary Table 11** for the results.

We conducted a subgroup analysis based on the regions where the included studies were conducted. Among these, three articles (61, 63, 64) investigated the prevalence of total hip bone loss in patients with systemic lupus erythematosus in Europe, involving a total of 249 subjects, with considerable heterogeneity across the studies (I² = 55.8%). The results showed a prevalence of 38.10% [95% CI (32.2%, 44.1%), P < 0.01], with a statistically significant difference; one study (62) investigated the prevalence of total hip bone loss in patients with systemic lupus erythematosus in Asia, involving a total of 125 subjects. The results showed a prevalence of 36.10% [95% CI (27.5%, 44.6%), P < 0.01]; one study (24) investigated the prevalence of total hip bone loss in patients with systemic lupus erythematosus in North America, involving a total of 89 subjects. The results showed a prevalence of 33.70% [95% CI (23.9%, 43.5%), P < 0.01]; see **Supplementary Figure 17** and **Supplementary Table 11** for the results.

Overall, within the paired data included in this study, the femoral neck was the site with the highest risk of bone loss in SLE patients. In terms of regional distribution, the point estimates of prevalence across different sites did not exhibit a consistent pattern of significant differences between regions, with most estimates being relatively close. As with the OP analysis, it must be clearly noted that the subgroup analyses for Asia and North America were based on data from a single study each, whilst the European subgroup was based on three studies. This severe imbalance in sample size means that the minor differences in point estimates currently observed do not have clear comparative significance. These results provide a preliminary description of the distribution based on the limited data available and do not yet support the conclusion that there are significant regional differences in prevalence. Further large-scale studies are required to validate the epidemiological characteristics of SLE-associated bone loss.

### Sensitivity analysis and publication bias of the prevalence of bone loss in SLE across different sites

In this study, the pooled analysis of the prevalence of bone loss across all skeletal sites (lumbar spine, femoral neck, total hip) showed low heterogeneity between studies. This indicates good consistency among the results of the included studies.

Funnel plots were used to assess publication bias. Funnel plots were constructed for the prevalence of bone loss in SLE stratified by anatomical site; the Egger’s test statistic for the lumbar spine was 0.078, the Egger’s test value for the femoral neck was 0.078, and the Egger’s test value for the total hip was 0.243. Consequently, there was no significant publication bias in the included literature; the results are shown in **Supplementary Figures 18**-**20**.

### Risk factors for OP in SLE

We also conducted a meta-analysis of risk factors for SLE complicated by OP to comprehensively identify risk indicators for this condition.

#### Sex

Three studies (65–67) investigated the association between gender and OP in patients with SLE. The heterogeneity among these studies was low (I²= 41.1%, P = 0.183). Utilizing a fixed-effect model, the analysis demonstrated that gender constitutes a risk factor for OP in SLE patients (OR 1.52, 95% CI 1.03, 2.25, P = 0.037). Further gender-specific analysis indicated that male SLE patients exhibited a higher risk of OP (OR 1.78, 95% CI 0.78, 4.08, P = 0.171), while female SLE patients exhibited a higher risk of OP (OR 1.45 95% CI 0.93, 2.27, P = 0.103). Although these results suggest a trend, the differences did not reach statistical significance. These findings do not entirely correspond with previous prevalence studies. When integrating prevalence data, it appears that premenopausal women may have a slightly lower risk of OP compared to men, whereas postmenopausal women display a significantly increased risk. In males, the risk does not appear to vary significantly across different age groups. Nevertheless, the overall risk of OP increases with age in both genders. Due to the substantial heterogeneity among risk factors, a sensitivity analysis was performed to identify sources of this heterogeneity (see **Supplementary Figure 21**). Notably, excluding the study by Eviatar et al. (66) led to a significant alteration in the pooled effect size (OR = 2.31), which reflects recent treatment trends. Temporal differences may introduce period-effect heterogeneity (refer to **Supplementary Figure 22**). Publication bias was evaluated with a funnel plot, which was specifically constructed to assess gender as a risk factor for OP in SLE. The Egger’s test yielded a value of 0.13, suggesting no significant publication bias in the studies included. The results are detailed in **Supplementary Figure 23** and **Table 3**.

**Table 3:** Summary of risk factors for OP in SLE.

Subgroup category	Specific classification	Number of studies within this subgroup	Total sample size(n)	Heterogeneity I2	Effect size OR(95%CI)	P Value	Egger
Sex	Total	3	/	41.1%	1.52(1.03, 2.25)	0.037	0.13
	Male	1	/	/	1.78(0.78, 4.08)	0.171	
	Female	2	/	68.8%	1.45(0.93, 2.27)	0.103	
Age	Total	8	/	88.3%	1.06(1.02, 1.1)	P<0.01	0.614
Age Group							
	Age>50 Only	1	125	/	21.92(7.19, 66.89)	P<0.01	
	All Age Adults	7	/	81.1%	1.06(1.03, 1.09)	P<0.01	
Area							
	Asia	5	/	90.2%	1.09(0.99, 1.2)	0.076	
	North America	1	/	/	1.06(1.03, 1.08)	P<0.01	
	Africa	1	902	/	1.03(1.01, 1.05)	P<0.01	
	Europe	1	110	/	1.06(1.02, 1.1)	P<0.01	
GCs	Total	12	/	92.3%	1.63(1.41, 1.89)	P<0.01	0
Area							
	Asia	10	/	93.4%	1.77(1.48, 2.12)	P<0.01	
	North America	1	/	/	1.6(1.07, 2.41)	0.023	
	Africa	1	902	/	1.16(1.08, 1.26)	P<0.01	
Participants							
	Both sexes	10	/	90.6%	2.05(1.49, 2.83)	P<0.01	
	Females only	1	155	/	1.05(1.01, 1.09)	0.012	
	Pre-menopausal women	1	60	/	1.06(1.01, 1.11)	0.016	
	Total	5	46225	75.9%	2.28(1.65, 3.16)	P<0.01	0.551
GC dose							
	≤ 10 mg/day	3	23649	51.3%	2.18(1.61, 2.94)	P<0.01	
	> 10 mg/day	2	22576	91.6%	2.39(1.03, 5.55)	0.044	
Course of disease in SLE patients	Total	4	/	76.7%	1.05(1, 1.1)	0.03	0.675
Area							
	North America	1	/	/	0.96(0.89, 1.04)	0.365	
	Africa	1	902	/	1.03(1, 1.07)	0.043	
	Asia	2	1198	11.9%	1.09(1.05, 1.12)	P<0.01	
SLEDAI	Total	4	/	93.8%	0.97(0.9, 1.05)	0.467	0.765
Area							
	Asia	2	/	93.2%	0.6(0.27, 1.32)	0.204	
	North America	1	/	/	1.00(1, 1.01)	0.029	
	Africa	1	902	/	1.07(1.04, 1.1)	P<0.01	

#### Age

Eight studies (13, 39, 55, 61, 65–68) have evaluated the relationship between age and OP in individuals diagnosed with SLE. The studies demonstrated significant heterogeneity (I²=88.3%, P<0.01). Utilizing a random-effects model, the analysis indicated a statistically significant association between age and OP (OR 1.06, 95% CI 1.02, 1.1, P<0.01). The findings suggest that SLE patients aged over 50 years’ experience an approximately 21-fold increased risk of developing OP (OR 21.92, 95% CI 7.19,66.89, P<0.01). Therefore, SLE patients aged 50 years and older represent a high-risk group for OP, with significantly elevated incidence rates compared to younger cohorts. These results are detailed in **Supplementary Figure 24** and **Table 3**.

Within the age subgroups, we conducted additional analyses stratified by the geographical location of the studies. Five studies (13, 55, 66–68) evaluated the association between SLE and OP within Asian populations. These analyses utilized random-effects models, which indicated considerable heterogeneity among the studies (I²=90.2%, P<0.01). The findings demonstrated no statistically significant association between SLE and an increased risk of OP in the Asian population (OR 1.09, 95% CI 0.99,1.2, P = 0.076). Conversely, one study (65) investigated this association in North American populations, identifying a 6% increased risk of OP among North Americans compared to other populations (OR 1.06, 95% CI 1.03,1.08, P<0.01), with the difference being statistically significant. One study (39) assessed the association between SLE and OP in African populations, revealing a 3% increased risk of OP in African individuals compared to other populations (OR 1.03, 95% CI 1.01,1.05, P<0.01), representing a statistically significant difference; One study (61) assessed the association between SLE and OP in European populations, revealing a 6% increased risk of OP in Europeans compared to other populations (OR 1.06, 95% CI 1.02,1.10, P<0.01), with the difference being statistically significant. In summary, the incidence risk among European and North American populations is relatively similar, a finding consistent with prevalence data. This may be attributed to the historical predominance of European immigrants in North America, resulting in comparable ethnic profiles. African populations exhibit a lower risk. Consequently, clinicians should pay heightened attention to the risk of OP in SLE patients from Europe and North America. Results are presented in **Supplementary Figure 25** and **Table 3**.

Due to the substantial heterogeneity observed among the included articles, a sensitivity analysis was conducted to identify potential sources of this variability. The exclusion of the study by Yee et al. (13) led to a significant alteration in the pooled effect size (OR = 1.07). Given that this study was published prior to the other included studies, which were all published post-2014, the year of publication may constitute a source of heterogeneity. The results are detailed in **Supplementary Figure 26**.

Publication bias was evaluated through a funnel plot analysis, focusing on age as a risk factor for OP in individuals with SLE. The Egger’s test yielded a value of 0.614, suggesting the absence of significant publication bias among the studies included in the analysis. Detailed results can be found in **Supplementary Figure 27**.

#### GCs

Twelve studies (25, 39, 46, 65–69) investigated the relationship between GC use and OP in patients with SLE. The studies exhibited significant heterogeneity (I²=92.3%, P<0.01). Utilizing a random-effects model, the analysis demonstrated a 63% increased risk of OP in SLE patients undergoing GC treatment, with the difference being statistically significant (OR 1.63, 95% CI 1.41, 1.89; P<0.01). Detailed results are provided in **Supplementary Figure 28** and **Table 3**.

In the subgroup of patients undergoing GC therapy, we performed a further subgroup analysis stratified by the geographical region of the studies. Ten studies (25, 46, 66–69) evaluated the association between GC use and OP in patients with SLE within Asian regions. A significant degree of heterogeneity was observed among these studies (I²=93.4%, P<0.01). Utilizing a random-effects model, the findings revealed a 77% increased risk of OP in SLE patients receiving GCs in Asia-based studies (OR 1.77, 95% CI 1.48,2.12, P<0.01), indicating a statistically significant difference. Additionally, one study examined the association between GC use and OP in SLE patients in North America (65), revealing a 60% increased risk of OP (OR 1.6, 95% CI 1.07,2.41, P = 0.023), which was also statistically significant. Furthermore, one study investigated this association in the African region (39).The results demonstrated a 16% increased risk of OP among SLE patients receiving GCs in the African region (OR 1.16, 95%CI 1.08,1.26, P<0.01), with this difference reaching statistical significance. In summary, this subgroup analysis suggests that GC use significantly elevates the risk of concurrent OP in SLE patients across various geographical regions. However, the extent of this increased risk varies considerably by region, with the highest risk observed in Asia, followed by North America and Africa. This variation may be attributed to a complex interplay of factors such as genetic predisposition, levels of sunlight exposure, and regional prescribing practices. It is important to note that while Asia has contributed a substantial number of studies, these studies exhibit significant heterogeneity, whereas evidence from North America and Africa is relatively sparse. Therefore, although the conclusion that GCs are a significant risk factor for OP in SLE patients is universally applicable, the precise magnitude of this risk may differ geographically. Future high-quality, multi-regional studies are necessary to elucidate the specific factors contributing to these regional differences. Detailed results are provided in **Supplementary Figure 28** and **Table 3**.

Within the subgroup of patients undergoing GC therapy, we conducted further subgroup analyses based on the populations included in the studies. Ten studies (39, 65–69) evaluated the association between GC use and OP in mixed-sex cohorts of patients with SLE. The studies exhibited significant heterogeneity (I²=90.6%, P<0.01), and analyses were performed using a random-effects model. The findings demonstrated a 1.05-fold increase in the risk of OP among SLE patients receiving GCs in the mixed-gender cohort (OR 2.05, 95%CI 1.49,2.83, P<0.01), indicating a statistically significant difference. One study (25) investigated the association between GC use and OP in female SLE patients, revealing a 5% increased risk of OP in this group (OR 1.05, 95% CI 1.01,1.09, P = 0.012), which was statistically significant. Additionally, one study (46) examined the association between GC use and OP in premenopausal women with SLE, showing a 6% increased risk of OP in premenopausal SLE patients receiving GCs (OR 1.06, 95% CI 1.01,1.11, P = 0.016), with the difference being statistically significant. In conclusion, this subgroup analysis of patients with SLE undergoing GC treatment reveals a significant association between GC use and an elevated risk of OP in this population. Notably, the analysis indicates that female SLE patients on GCs may not demonstrate an increased risk of OP; however, this finding should be approached with caution due to the limited number of studies included. Detailed results are provided in **Supplementary Figure 29** and **Table 3**.

Within the subgroup of patients treated with GCs, we conducted further analyses stratified by varying GC dosages. Three studies (66, 69) evaluated the relationship between GC doses of ≤10 mg per day and the incidence of OP(OP) in patients with SLE. These studies exhibited substantial heterogeneity (I²=51.3%, P = 0.128). Utilizing a random-effects model, the findings demonstrated that SLE patients receiving daily GC doses of ≤10 mg had a 1.18-fold increased risk of developing OP (OR 2.18, 95%CI 1.61,2.94, P<0.01), indicating a statistically significant association. Conversely, two studies (69) investigated the association between GC doses exceeding 10 mg per day and the risk of OP in SLE patients, revealing significant heterogeneity (I²=91.6%, P<0.01).An analysis employing a random-effects model demonstrated that SLE patients administered GCs at doses exceeding 10 mg per day exhibited a 1.39-fold increased risk of concurrent OP(OR 2.39, 95% CI 1.03,5.55, P<0.01), indicating a statistically significant difference. These findings corroborate previous research suggesting that GCs have a detrimental impact on bone density. Therefore, it is imperative for SLE patients to exercise caution when modifying GC dosages. Extended high-dose GC therapy requires vigilant monitoring of bone density to mitigate the adverse effects of OP and improve patients’ quality of life. The results are detailed in **Supplementary Figure 30** and **Table 3**.

Due to the significant heterogeneity observed among studies examining risk factors associated with GC use, a sensitivity analysis was conducted to identify potential sources of this variability. Notably, the exclusion of the study by Jung et al. (25) led to a marked alteration in the pooled effect size (OR = 1.85). Given that this particular study included women from a wide range of age groups, the demographic composition of the studies may contribute to the observed heterogeneity. The results of this analysis are detailed in **Supplementary Figure 31**.

A funnel plot was utilized to evaluate publication bias in the context of risk factors for SLE associated with OP in patients undergoing GC treatment. The analysis demonstrated asymmetry on both sides of the funnel plot, with an Egger’s test yielding a value of 0, indicating the presence of publication bias in the studies under review. The findings are illustrated in **Supplementary Figure 32**.

### Course of disease in SLE patients

Four studies (39, 65–67) evaluated the relationship between the duration of SLE and the incidence of OP among affected patients. The studies exhibited significant heterogeneity (I²=76.7%, P<0.01), prompting the use of a random-effects model for analysis. The findings suggest that prolonged SLE duration is associated with a 5% increased risk of developing OP (OR 1.05, 95% CI 1.00,1.10, P = 0.03). Detailed results can be found in **Supplementary Figure 33** and **Table 3**.

Within the subgroup analysis of SLE disease course, we further stratified the analyses by the geographical location of the studies. Two studies (66, 67) evaluated the relationship between the disease course and the incidence of OP among Asian SLE patients, revealing minimal heterogeneity (I²=11.9%, P = 0.287). The findings demonstrated that the duration of the disease in Asian SLE patients was associated with a 9% increased risk of OP (OR 1.09, 95% CI 1.05,1.12, P<0.01). In contrast, one study (65) examined the association between disease duration and OP occurrence in North American SLE patients, indicating a 4% reduction in OP risk (OR 0.96, 95% CI 0.89,1.04, P = 0.365). Additionally, another study (39) investigated this association in African SLE patients, showing a 3% increased risk of OP (OR 1.03, 95% CI 1.00,1.07, P = 0.043). These results are detailed in **Supplementary Figure 33** and **Table 3**.

Due to the substantial heterogeneity observed among the included articles, a sensitivity analysis was performed to ascertain the sources of this variability. The exclusion of the study by Davidson et al. (65) led to a significant alteration in the pooled effect size (OR = 1.07). Given that this particular study encompassed women from all age groups, the inclusion criteria of the studies may contribute to the observed heterogeneity. The results are detailed in **Supplementary Figure 34**.

A funnel plot was utilized to evaluate publication bias, and the Egger’s test yielded a value of 0.675, suggesting an absence of significant publication bias among the studies included. The results are detailed in **Supplementary Figure 35**.

### SLEDAI

Four studies (25, 39, 65, 68) investigated the relationship between the SLE Disease Activity Index (SLEDAI) and OP in individuals diagnosed with SLE. The studies exhibited significant heterogeneity (I²=93.8%, P<0.01), warranting the application of a random-effects model for analysis. The findings suggested that SLEDAI was associated with a 3% reduction in the risk of OP among SLE patients (OR 0.97, 95% CI 0.90,1.05, P = 0.467). These results are detailed in **Supplementary Figure 36** and **Table 3**.

Within the SLEDAI subgroup, a further analysis was conducted based on the geographical location of the studies. Two studies (25, 68) examined the association between SLEDAI and OP in Asian patients with SLE, revealing substantial heterogeneity between the studies (I²=93.2%, P<0.01). The application of a random-effects model indicated that SLEDAI was associated with a 40% reduction in the risk of OP among Asian SLE patients (OR 0.6, 95% CI 0.27,1.32, P = 0.204), although this finding was not statistically significant. In contrast, a study (65) focusing on North American SLE patients reported no significant change in OP risk (OR 1.0, 95% CI 1, 1.01, P = 0.029), with the result being statistically significant. Additionally, a study (39) investigating African SLE patients found a 7% increase in OP risk associated with SLEDAI (OR 1.07, 95% CI 1.04,1.10, P<0.01), which was statistically significant. This finding is significant because only studies conducted in Africa have shown an increased risk of OP associated with high SLE activity, whereas no statistically significant differences were observed in other regions. Consequently, future research should prioritize investigations within African populations to further explore ethnic differences across regions. The results are detailed in **Supplementary Figure 36** and **Table 3**.

Due to the substantial heterogeneity observed among the included articles, a sensitivity analysis was performed to ascertain the sources of this variability. Notably, the exclusion of the study by Gamal et al. (39) led to a significant alteration in the pooled effect size (OR = 0.76). This variation may be attributed to the study’s African context, which contrasts with the geographical settings of the other studies and may contribute to the observed heterogeneity. The results of this analysis are detailed in **Supplementary Figure 37**.

Funnel plots were utilized to evaluate publication bias. Specifically, a funnel plot was generated to examine age as a risk factor for SLE in relation to OP. The Egger’s test yielded a value of 0.765, suggesting the absence of significant publication bias among the studies included. The results are detailed in **Supplementary Figure 38**.

### Risk factors for low bone mineral density in SLE

#### GCs

Four studies (13, 31, 60, 70) evaluated the association between GC use and low bone mineral density in patients with SLE. Notable heterogeneity was observed among the studies (I²=86.7%, P<0.01). Utilizing a random-effects model for analysis, the findings revealed a 61% increased risk of low bone mineral density in SLE patients undergoing GC treatment (OR 1.61, 95% CI 1.05, 2.46, P = 0.028). The results are detailed in **Supplementary Figure 39** and **Table 4**.

**Table 4:** Summary of risk factors for low bone mineral density in SLE.

Subgroup category	Specific classification	Number of studies within this subgroup	Total sample size(n)	HeterogeneityI2	Effect size OR(95%CI)	P Value	Egger
GCs	Total	4	672	86.7%	1.61(1.05, 2.46)	0.028	0.026
Area							
	North America	1	91	/	1.04(1.01, 1.07)	P<0.01	
	South America	1	211	/	3.97(1.51, 10.41)	P<0.01	
	Asia	2	370	0%	1.64(1.3, 2.06)	P<0.01	
Menopause	Total	3	242	40%	5(2.46, 10.14)	P<0.01	0.902

Within the GC subgroup, a further subgroup analysis was performed based on the geographical region of the studies. Two studies (13, 60) evaluated the association between GC use and low bone mineral density among patients with SLE in the Asian region. The studies exhibited minimal heterogeneity (I²=0%, P<0.01). The findings demonstrated a 64% increased risk of low bone mineral density in SLE patients receiving GCs in Asian-based studies (OR 1.64, 95% CI 1.3,2.06, P<0.01), indicating a statistically significant difference. In North America, one study (31) investigated this association and found a 4% increased risk of low bone mineral density in SLE patients on GCs (OR 1.04, 95% CI 1.01,1.07, P<0.01), also indicating statistical significance. Additionally, one study (70) conducted in South America reported a 2.97-fold increased risk of low bone mineral density in SLE patients using GCs. The results are detailed in **Supplementary Figure 39** and **Table 4**.

Due to the substantial heterogeneity observed among the included studies, a sensitivity analysis was conducted to ascertain the sources of this variability. Notably, the exclusion of the study by Cramarossa et al. (31) led to a significant alteration in the pooled effect size (OR = 1.98). This change may be attributed to the potential genetic predispositions present in Asian and South American populations, which may increase their susceptibility to GC-induced bone loss. The results of this analysis are detailed in **Supplementary Figure 40**.

A funnel plot was utilized to evaluate publication bias in the context of risk factors associated with SLE complicated by low bone mineral density subsequent to GC use. The Egger’s test yielded a value of 0.026, suggesting the presence of publication bias among the included studies. The results are depicted in **Supplementary Figure 41**.

#### Menopause

Three studies (40, 55, 71) evaluated the relationship between menopause and reduced bone mineral density in individuals with SLE. The heterogeneity among these studies was low (I²= 40%, P = 0.189). Utilizing a fixed-effect model, the analysis revealed a fourfold increase in the risk of low bone mineral density among menopausal SLE patients (OR 5, 95% CI 2.46,10.14, P<0.01), with this difference reaching statistical significance. The findings are detailed in **Supplementary Figure 42** and **Table 4**.

A funnel plot was utilized to examine publication bias concerning the risk factor of low bone mineral density in postmenopausal women with SLE. The Egger’s test yielded a value of 0.9, indicating no significant publication bias in the studies included. These results are depicted in **Supplementary Figure 43**.

## Discussion

### Key findings

This study, through a systematic review and meta-analysis, comprehensively assessed the prevalence of OP and osteopenia in patients with SLE, as well as regional variations and key influencing factors. The results indicate that the overall prevalence of OP in SLE patients is 16.70%, which is significantly higher than that in the general population, highlighting the serious challenges this group faces in terms of bone health. Site-specific analysis indicated that lumbar spine OP (10.0%) was the most commonly affected site, with a higher prevalence than the femoral neck and total hip. The most common site of osteopenia was the femoral neck, with a prevalence of 44.1%, higher than that of the lumbar spine and total hip. This seemingly inconsistent pattern actually reveals pathophysiological clues regarding the dynamic progression of SLE-related bone disease: the high rate of osteopenia in the femoral neck reflects an early, diffuse negative bone balance caused by the SLE disease state (such as chronic inflammation), which may be related to its role as a primary weight-bearing site and serves as a universal warning sign of increased fracture risk (72, 73); Conversely, the lumbar spine, being a site rich in trabecular bone and highly sensitive to glucocorticoids and inflammatory factors, progresses preferentially and more severely to OP when exposed to defined risks (particularly medium-to-high-dose glucocorticoids), becoming the most vulnerable ‘target’ in the skeleton during the later stages of the disease (74, 75). Therefore, in clinical practice, a comprehensive assessment should be conducted by combining DXA results from these two sites: results from the femoral neck emphasize the importance of early, comprehensive skeletal health management and fracture prevention; whilst a diagnosis of OP in the lumbar spine is a key indication for initiating active anti-OP drug therapy.” Furthermore, this study identified several independent risk factors for SLE-associated OP, including advanced age (particularly >50 years), postmenopausal status, long-term or high-dose glucocorticoid use, and a longer duration of SLE. It is worth noting that the association between glucocorticoid use and lumbar vertebral fractures was particularly pronounced, whereas its impact on the femoral neck and total hip was relatively weaker, suggesting that different skeletal sites may exhibit varying degrees of sensitivity to glucocorticoids.

### Comparison with other studies of the same type

Our research, through quantitative analysis, has for the first time unequivocally revealed the magnitude and patterns of regional differences, elevating previous anecdotal observations to conclusive evidence, all confirming that SLE patients constitute a high-risk group for OP. However, this study offers the advantage of providing the most precise global prevalence estimates to date through quantitative synthesis of a large sample size. Compared to earlier reviews, we conducted the first detailed meta-analysis of prevalence across different skeletal sites, revealing the high susceptibility of the lumbar spine. This finding provides direct evidence for prioritizing clinical monitoring of this region. Regarding risk factors, our analysis of GC dosage (with a significant increase in risk at daily doses exceeding 10mg) aligns with clinical guidelines on GC-induced OP. However, this study elevates the evidence level to that of a systematic review within the specific SLE population. Compared to the study by Gu et al. (76), our analysis incorporated more recent studies and conducted more in-depth subgroup analyses by region and population. Some findings, such as the higher prevalence of lumbar OP in Asian populations, were reported for the first time. The study by Ji et al. (77) subsequently corroborated this observation.

### Implications of diagnostic criteria variability

The subgroup analysis revealing a differential prevalence of OP under the WHO criteria (T-score ≤ -2.5; 15.7%) versus other diagnostic criteria (20.8%) is not merely a methodological footnote. This finding carries substantial clinical and research significance. Clinically, it underscores that the reported burden of OP in SLE is sensitive to the diagnostic threshold applied, highlighting the risk of under-diagnosis with overly strict criteria or over-diagnosis with less stringent ones. It argues for the standardized application of WHO DXA criteria to ensure consistent patient identification (78). From a research perspective, this heterogeneity explains, in part, the wide range of prevalence estimates reported in the literature. It serves as a critical reminder for the interpretation and comparison of epidemiological studies, emphasizing the need to account for diagnostic variability when synthesizing evidence. Therefore, our analysis not only quantifies the prevalence but also provides a key to reconciling discrepancies across previous studies.

### Mechanism analysis

The high incidence of OP and reduced bone mass in SLE patients results from the combined effects of multiple factors. Firstly, prolonged GC use constitutes one of the core mechanisms, accelerating bone loss through multiple pathways including promoting osteoclast activation, inhibiting osteoblast function, and inducing osteocyte apoptosis. Findings from this study provide robust support for this mechanism (79–81).

Secondly, the chronic inflammatory state inherent to SLE itself is crucial. Multiple inflammatory cytokines, such as tumor necrosis factor-α (TNF-α), interleukin-1 (IL-1), and interleukin-6 (IL-6), play key roles in SLE. These factors not only directly stimulate osteoclast generation but also inhibit bone formation, resulting in abnormal high-turnover bone metabolism (82–84).

Additionally, alterations in sex hormone levels, particularly the sharp decline in estrogen following menopause, eliminate crucial protective effects on bone. This aligns closely with the significantly elevated risk observed in postmenopausal women in this study (85). Furthermore, vitamin D deficiency is highly prevalent in SLE patients, associated with disease activity and photosensitivity, and may be exacerbated by renal involvement, further impairing calcium absorption and bone metabolism (86). Finally, renal involvement frequently manifests as lupus nephritis (LN), one of SLE’s most severe complications. Research indicates that lupus nephritis not only impacts renal function but may also affect bone health through mechanisms such as secondary hyperparathyroidism (87, 88). Secondary hyperparathyroidism is not uncommon in SLE patients, and this pathological state may lead to increased risk of OP and fractures (89). The extended disease duration identified in this study as a risk factor also indirectly supports the pathogenic role of chronic inflammation and cumulative organ damage.

Therefore, the pathogenesis of SLE-associated bone disease is not uniform but exhibits population-specific nuances driven by the interplay of the aforementioned mechanisms. For instance, the markedly elevated risk in postmenopausal women stems from the convergence of estrogen-deprived bone loss and chronic inflammatory/GC-mediated damage (79–85). Similarly, the potential heightened susceptibility of the lumbar spine in Asian populations may reflect an interaction between genetic or constitutional factors (e.g., differences in baseline bone geometry or vitamin D metabolism) and the particular sensitivity of trabecular-rich bone to GCs (90–93). Crucially, the prevalent state of osteopenia (39.5%) represents an active phase in this pathological continuum, not a static condition. Progression to OP is fueled by persistent drivers: unchecked inflammation, ongoing GC exposure, and worsening vitamin D deficiency or renal dysfunction. This transition is characterized not merely by quantitative bone loss but by accelerated deterioration of bone microarchitecture, significantly increasing fracture risk. This understanding mandates a shift in clinical strategy: from solely diagnosing OP to actively intercepting its progression in high-risk subgroups (e.g., postmenopausal women, Asian patients, GC users) during the osteopenic stage, with targeted monitoring and early intervention.

### Clinical implications of this study

The findings of this study provide clear guidance for the clinical management of systemic lupus erythematosus. Firstly, they strongly support the use of combined dual-energy X-ray absorptiometry (DXA) of the lumbar spine and femoral neck as the standard protocol for assessing bone health in patients with SLE. This strategy enables the simultaneous achievement of early risk screening (via the femoral neck) and the determination of clear treatment indications (via the lumbar spine), thereby overcoming the limitations of single-site assessment and optimizing clinical decision-making pathways. Secondly, the core high-risk subgroups identified in the study (glucocorticoid users, postmenopausal women and elderly patients) provide a clear focus for implementing targeted and effective bone health monitoring. Clinical resources should be prioritized for these groups, with the establishment of more intensive follow-up and earlier intervention protocols. Crucially, this study found that nearly 40% of SLE patients have osteopenia, revealing a significant preventive window that has been under-recognized in current clinical practice. This necessitates a shift in mindset among rheumatologists, moving from the passive diagnosis of ‘OP’ to the active management of ‘osteopenia’, and initiating comprehensive management strategies—including lifestyle interventions, risk factor control and, where necessary, pharmacological prevention—for high-risk patients before fractures occur.

In summary, this study provides key evidence for establishing a new paradigm for skeletal health management in SLE patients, based on risk stratification, combined site assessment and phased proactive intervention. Its core objective is to achieve a paradigm shift from ‘treating fractures’ to ‘preventing fractures’, ultimately improving patients’ long-term prognosis.

### Limitations and improvement measures

This study has the following limitations. Firstly, there was significant heterogeneity among the included studies. Although we explored some sources of this through subgroup and sensitivity analyses, differences in unreported factors (such as detailed baseline characteristics and lifestyle factors) remain a major source of variation. Secondly, publication bias may be present, which could lead to an overestimation of prevalence. Thirdly, the limited number of studies included in certain subgroup analyses (e.g., postmenopausal women, specific geographical regions) undermines the robustness of the results. Fourthly, although glucocorticoid (GC) use, disease duration and immunosuppressive therapy are considered key factors influencing bone loss in SLE patients, this study did not include them as primary variables in the subgroup analyses. This is primarily because the vast majority of the included original studies failed to report detailed exposure data on GC (such as precise cumulative doses and duration of treatment) and clear disease duration in a systematic and consistent manner; there was also a general lack of standardized descriptions regarding the use of disease-modifying antirheumatic drugs and biologics. This widespread absence of raw data makes meaningful quantitative pooling and comparison at this level methodologically unfeasible. Fifth, the analysis of risk factors was primarily based on observational studies, making it difficult to establish clear causal relationships.

To address these gaps in the evidence, future research should be advanced on multiple fronts. At the data collection level, there is an urgent need for rigorously designed prospective cohort studies to standardize and prospectively collect precise cumulative GC doses, treatment duration, details of concomitant medications, and accurate disease progression; this is a prerequisite for any in-depth analysis. At the analytical level, based on high-quality data, future research should quantify the dose-time-effect relationship of GCs, assess the independent contribution of disease course, and identify the potential independent effects (protective or harmful) of various DMARDs and biologics on bone health. At the study population level, targeted research should be strengthened for postmenopausal women, male patients, and underrepresented groups. At the methodological level, approaches such as Mendelian randomization can be employed to strengthen causal inference; randomized controlled trials should be conducted to evaluate the efficacy of preventive strategies; and the integration of biomarkers should be explored to construct precise risk prediction models. By systematically pursuing these directions, future research is expected to provide a robust and actionable evidence base for establishing proactive skeletal health management strategies for SLE patients based on individual risk.

## Conclusion

Our research indicates that postmenopausal women, Asian patients, and individuals undergoing long-term glucocorticoid therapy constitute key populations for OP screening in SLE. We recommend implementing ‘site-specific monitoring’ (with particular emphasis on the lumbar spine) alongside ‘proactive bone protection strategies’. These measures should commence at the initiation of glucocorticoid treatment, irrespective of dosage.

## Data Availability

The original contributions presented in the study are included in the article/**Supplementary Material**. Further inquiries can be directed to the corresponding authors.
