# Supplementary material for: Global prevalence, site-specific patterns, and key risk factors for osteoporosis and bone loss in systemic lupus erythematosus: a systematic review and meta-analysis
Source: Front Immunol. 2026 Apr 15;17:1778825. doi: 10.3389/fimmu.2026.1778825 (PMC13125140; doi:10.3389/fimmu.2026.1778825)
Supplement: Supplementary file 1 [file SupplementaryFile1.docx]

**SUPPLEMENTARY MATERIAL**

**Table of Contents**

**Methods 1:Search Strategy 1**

**Table S1:The overall prevalence of osteoporosis in patients with systemic lupus erythematosus 3**

**Table S2:The prevalence of SLE in patients with osteoporosis across different anatomical sites. 6**

**Table S3:Factors Influencing Osteoporosis and Low Bone Mineral Density in Systemic Lupus Erythematosus 7**

**Table S4:Newcastle-Ottawa Quality Assessment Form for Cohort Studies 9**

**Table S5:Cross-Sectional/Prevalence Study Quality 12**

**Table S6:Prevalence of osteoporosis in the lumbar spine 14**

**Table S7:Prevalence of osteoporosis in the femoral neck 14**

**Table S8:Prevalence of osteoporosis in the total hip 14**

**Table S9:Prevalence of osteopenia in the lumbar spine 15**

**Table S10:Prevalence of osteopenia in the femoral neck 15**

**Table S11:Prevalence of osteopenia in the total hip 15**

**Figure S1:Meta-regression of geographic region on the prevalence of osteoporosis in the “Other Diagnostic Criteria” subgroup 16**

**Figure S2:Meta-regression of participant type (e.g., pre-/post-menopausal) on the prevalence of osteoporosis in the “Other Diagnostic Criteria” subgroup 17**

**Figure S3:Meta-regression of participant type (e.g., pre-/post-menopausal) on the prevalence of osteoporosis in the “Other Diagnostic Criteria” subgroup 17**

**Figure S4:Meta-regression of sex composition on the prevalence of osteoporosis in the “Other Diagnostic Criteria” subgroup 18**

**Figure S5:Funnel plot: Overall Prevalence of Osteoporosis in Systemic Lupus Erythematosus. 19**

**Figure S6:Forest plot: Prevalence of lumbar osteoporosis in systemic lupus erythematosus, by study region. 19**

**Figure S7:Forest plot: Prevalence of osteoporosis at the femoral neck in systemic lupus erythematosus, by study region. 20**

**Figure S8:Forest plot: Prevalence of osteoporosis at the total hip in systemic lupus erythematosus, by study region. 20**

**Figure S9:Sensitivity analysis: Prevalence of osteoporosis in the lumbar spine in systemic lupus erythematosus. 21**

**Figure S10:Funnel plot: Prevalence of lumbar osteoporosis in systemic lupus erythematosus. 21**

**Figure S11:Funnel plot: Prevalence of osteoporosis at the femoral neck in systemic lupus erythematosus 22**

**Figure S12:Funnel plot: Prevalence of osteoporosis at the total hip in systemic lupus erythematosus. 22**

**Figure S13:Sensitivity analysis: Prevalence of osteopenia in Systemic Lupus Erythematosus. 23**

**Figure S14:Funnel plot: Prevalence of Osteopenia in Systemic Lupus Erythematosus. 23**

**Figure S15:Forest plot: Prevalence of osteopenia at the lumbar spine in systemic lupus erythematosus, by study region. 24**

**Figure S16:Forest plot: Prevalence of osteopenia at the femoral neck in systemic lupus erythematosus, by study region. 24**

**Figure S17:Forest plot: Prevalence of osteopenia at the total hip in systemic lupus erythematosus, by study region. 25**

**Figure S18:Funnel plot: Prevalence of osteopenia at the lumbar spine in systemic lupus erythematosus. 25**

**Figure S19:Funnel plot: Prevalence of osteopenia at the femoral neck in systemic lupus erythematosus. 26**

**Figure S20:Funnel plot: Prevalence of osteopenia at the total hip in systemic lupus erythematosus. 26**

**Figure S21:Forest plot:Risk factors for osteoporosis in systemic lupus erythematosus,by sex. 27**

**Figure S22:Sensitivity analysis:Risk factors for osteoporosis in systemic lupus erythematosus, by sex. 27**

**Figure S23:Funnel plot: Risk factors for osteoporosis in systemic lupus erythematosus, by sex. 28**

**Figure S24:Forest plot:Risk factors for osteoporosis in systemic lupus erythematosus,by age. 28**

**Figure S25:Forest Plot: Risk Factors for Osteoporosis in Systemic Lupus Erythematosus, Stratified by Age and Further Subgrouped According to Geographic Region. 29**

**Figure S26:Sensitivity analysis:Risk factors for osteoporosis in systemic lupus erythematosus, by age. 29**

**Figure S27:Funnel plot: Risk factors for osteoporosis in systemic lupus erythematosus, by age. 30**

**Figure S28:Forest Plot: Risk Factors for Osteoporosis in Systemic Lupus Erythematosus, Stratified by glucocorticoid utilization rate and Further Subgrouped According to Geographic Region. 30**

**Figure S29:Forest Plot: Risk Factors for Osteoporosis in Systemic Lupus Erythematosus, Stratified by glucocorticoid utilization rate and Further Subgrouped by Study Population Characteristics. 31**

**Figure S30:Forest Plot: Risk Factors for Osteoporosis in Systemic Lupus Erythematosus, Stratified by glucocorticoid dosage. 31**

**Figure S31:Sensitivity analysis:Risk factors for osteoporosis in systemic lupus erythematosus, by glucocorticoid. 32**

**Figure S32:Funnel plot: Risk factors for osteoporosis in systemic lupus erythematosus, by glucocorticoid. 32**

**Figure S33:Forest Plot: Risk factors for osteoporosis in systemic lupus erythematosus, by disease duration and Further Subgrouped According to Geographic Region. 33**

**Figure S34:Sensitivity analysis: Risk factors for osteoporosis in systemic lupus erythematosus, by disease duration. 33**

**Figure S35:Funnel plot: Risk factors for osteoporosis in systemic lupus erythematosus, by disease duration. 34**

**Figure S36:Forest Plot: Risk factors for osteoporosis in systemic lupus erythematosus, by SLE Disease Activity Index (SLEDAI) and Further Subgrouped According to Geographic Region. 34**

**Figure S37:Sensitivity analysis: Risk factors for osteoporosis in systemic lupus erythematosus, by SLE Disease Activity Index (SLEDAI). 35**

**Figure S38:Funnel plot: Risk factors for osteoporosis in systemic lupus erythematosus, by SLE Disease Activity Index (SLEDAI). 35**

**Figure S39:Forest plot:Risk factors for Low Bone Mineral Density in systemic lupus erythematosus,by by glucocorticoid utilization rate and Further Subgrouped According to Geographic Region. 36**

**Figure S40:Sensitivity analysis: Risk factors for Low Bone Mineral Density in systemic lupus erythematosus,by glucocorticoid utilization rate. 36**

**Figure S41:Funnel plot: Risk factors for Low Bone Mineral Density in systemic lupus erythematosus,by glucocorticoid utilization rate. 37**

**Figure S42:Forest plot: Risk factors for Low Bone Mineral Density in systemic lupus erythematosus, by menopausal status. 37**

**Figure S43:Funnel plot: Risk factors for Low Bone Mineral Density in systemic lupus erythematosus, by menopausal status. 38**

**Supplementary Methods 1——Search Strategy**

| Databases（Searches run Sep 26,2025） | Results |
| --- | --- |
| Pubmed | 426 |
| Embase | 706 |
| Cochrane library | 91 |
| Web of Science | 464 |
| CNKI | 49 |
| Wan Fang | 174 |

**PubMed**

(((prevalence) OR (risk factors)) OR (influence factor)) AND (((((((Lupus erythematosus, systemic[MeSH Terms]) OR (systemic lupus erythematosus)) OR (lupus)) OR (lupus nephritis)) OR (lupus vasculitis)) OR (SLE)) AND (("Lupus erythematosus, systemic"[MeSH Terms] OR "systemic lupus erythematosus"[All Fields] OR "lupus"[All Fields] OR "lupus nephritis"[All Fields] OR "lupus vasculitis"[All Fields] OR "SLE"[All Fields]) AND ((("Osteoporosis"[Mesh]) OR ((((((((((((((((((((((Osteoporoses) OR (Osteoporosis, Age-Related)) OR (Osteoporosis, Age Related)) OR (Age-Related Osteoporosis)) OR (Age-Related Osteoporoses)) OR (Age Related Osteoporosis)) OR (Osteoporoses, Age-Related)) OR (Bone Loss, Age-Related)) OR (Age-Related Bone Loss)) OR (Age-Related Bone Losses)) OR (Bone Loss, Age Related)) OR (Bone Losses, Age-Related)) OR (Osteoporosis, Senile)) OR (Osteoporoses, Senile)) OR (Senile Osteoporoses)) OR (Senile Osteoporosis)) OR (Osteoporosis, Involutional)) OR (Osteoporosis, Post-Traumatic)) OR (Osteoporosis, Post Traumatic)) OR (Post-Traumatic Osteoporoses)) OR (Post-Traumatic Osteoporosis)) OR (Bone loss))))))

**Web of science**

TS = (Osteoporosis OR Osteoporoses OR Osteoporosis,

Age-Related OR Osteoporosis, Age Related OR Age-Related

Osteoporosis OR Age-Related Osteoporoses OR Age Related

Osteoporosis OR Osteoporoses, Age-Related OR Bone Loss,

Age-Related OR Age-Related Bone Loss OR Age-Related

Bone Losses OR Bone Loss, Age Related OR Bone Losses,

Age-Related OR Osteoporosis, Senile OR Osteoporoses,

Senile OR Senile Osteoporoses OR Senile Osteoporosis OR

Osteoporosis, Involutional OR Osteoporosis,

Post-Traumatic OR Osteoporosis, Post Traumatic OR

Post-Traumatic Osteoporoses OR Post-Traumatic

Osteoporosis OR Bone loss) AND TS= systemic lupus erythematosus OR TS= lupus OR TS= SLE OR TI=systemic lupus erythematosus OR AB= systemic lupus erythematosus OR TI= sle OR AB= sle OR TI= lupus OR AB= lupus AND TS =（prevalence OR risk factors OR influence factor）

**Embase**

(’decalciﬁcation, pathologic’/exp OR ’decalciﬁcation,

pathologic’ OR ’endocrine osteoporosis’/exp OR ’endocrine

osteoporosis’ OR ’osteoporotic decalciﬁcation’/exp OR

’osteoporotic decalciﬁcation’ OR ’pathologic

decalciﬁcation’/exp OR ’pathologic decalciﬁcation’ OR

’osteoporosis’/exp OR ’osteoporosis’) AND ('systemic lupus erythematosus'/exp OR 'disseminated lupus' OR 'disseminated lupus erythematosis' OR 'disseminated lupus erythematosus' OR 'lupus erythematosus disseminatus' OR 'lupus erythematosus, systemic' OR 'sle' OR 'systemic lupus erythematous') AND ('prevalence' OR 'risk factors' OR 'influence factor')

**Cochrane library**

((“Osteoporosis”[Mesh]) OR ((((((((((((((((((((((Osteoporoses) OR (Osteoporosis, Age-Related)) OR (Osteoporosis, Age Related)) OR (Age-Related Osteoporosis)) OR (Age-Related Osteoporoses)) OR (Age Related Osteoporosis)) OR (Osteoporoses, Age-Related)) OR (Bone Loss, Age-Related)) OR (Age-Related Bone Loss)) OR (Age-Related Bone Losses)) OR (Bone Loss, Age Related)) OR (Bone Losses, Age-Related)) OR (Osteoporosis, Senile)) OR (Osteoporoses, Senile)) OR (Senile Osteoporoses)) OR (Senile Osteoporosis)) OR (Osteoporosis, Involutional)) OR (Osteoporosis, Post-Traumatic)) OR (Osteoporosis, Post Traumatic)) OR (Post-Traumatic Osteoporoses)) OR (Post-Traumatic Osteoporosis)) OR (Bone loss))) AND (systemic lupus erythematosus OR lupus OR lupus nephritis OR lupus vasculitis OR SLE) AND (prevalence OR lupus OR risk factors OR influence factor)

**CNKI：**(SU=('系统性红斑狼疮' + '狼疮' + 'SLE') AND SU=('骨质疏松' + '骨量减少' + '骨丢失') AND SU=('患病率' + '发病率' + '流行率' + '风险因素' + '影响因素' + '危险因素'))

**Wan Fang：**(主题:("系统性红斑狼疮" OR "狼疮" OR "SLE") AND 主题:("骨质疏松" OR "骨量减少" OR "骨丢失") AND 主题:("患病率" OR "发病率" OR "流行率" OR "风险因素" OR "影响因素" OR "危险因素"))

# **Supplementary Table S1-S3:Demographic characteristics of included studies.**

**Supplementary Table S1: The overall prevalence of osteoporosis in patients with systemic lupus erythematosus**

| Author | Number of OP cases | Number of OPE cases | Number of SLE cases | area | Study type | participants | Diagnosis |
| --- | --- | --- | --- | --- | --- | --- | --- |
| Yee,2014[13] | 25 | NA | 242 | Europe | CS | Adult group | DXA |
| Yeap,2009[14] | 6 | 41 | 98 | Asia | CS | Premenopausal group | DXA |
| Toloza, 2010[15] | 12 | 28 | 117 | North America | CS | Adult group | DXA |
| Tang,2012[16] | 13 | 61 | 150 | Asia | CS | Adult group | DXA |
| Sun,2015[17] | 10 | 37 | 119 | Asia | CS | Adult group | DXA |
| Souto, 2012[18] | 18 | 37 | 68 | South America | CS | Postmenopausal females plus males aged > 50 years | DXA |
| Pineau, 2004[19] | 37 | 100 | 205 | North America | CS | Adult group | DXA |
| Mendoza-Pinto,2009（2）[20] | 37 | 106 | 210 | North America | CS | Adult group | DXA |
| Mendoza-Pinto, 2009（1）[21] | 5 | 40 | 100 | North America | CS | Premenopausal group | DXA |
| Li, 2010[22] | 6 | 31 | 59 | Asia | CS | Adult group | DXA |
| Li, 2009[23] | 33 | 58 | 152 | Asia | CS | Adult group | DXA |
| Lee, 2012[24] | 40 | NA | 89 | North America | CS | Postmenopausal group | DXA |
| Jung, 2020[25] | 51 | NA | 155 | Asia | CS | Adult group | DXA |
| Hansen, 2014[26] | 2 | 14 | 33 | Europe | CS | Adult group | DXA |
| Guo, 2017[27] | 16 | 37 | 60 | Asia | CS | Adult group | DXA |
| García-Carrasco, 2017[28] | 10 | 28 | 110 | North America | Cohort Study | Adult group | DXA |
| Furukawa, 2013[29] | 7 | 26 | 52 | Asia | CS | Adult group | DXA |
| Fischer-Betz, 2005[30] | 248 | NA | 1033 | Europe | CS | Adult group | Unspecified |
| Cramarossa, 2017[31] | 11 | NA | 91 | North America | CS | Postmenopausal females plus males aged > 50 years | DXA |
| Bultink, 2005[32] | 4 | 42 | 107 | Europe | CS | Adult group | DXA |
| Boyanov, 2003[33] | 22 | 6 | 32 | Europe | Cohort Study | Adult group | DXA |
| Chong, 2007[34] | 4 | 28 | 60 | Asia | CS | Premenopausal group | DXA |
| Almehed, 2007[35] | 37 | 62 | 163 | Europe | CS | Adult group | DXA |
| Correa-Rodríguez, 2021[36] | 10 | 63 | 121 | Europe | CS | Adult group | DXA |
| Shevchuk, 2020[37] | 7 | 19 | 38 | Europe | CS | Postmenopausal group | DXA |
| Korczowska, 2003[38] | 8 | 15 | 38 | Europe | CS | Adult group | Unspecified |
| Gamal, 2023[39] | 80 | NA | 902 | Africa | Cohort Study | Adult group | Unspecified |
| Furukawa, 2011[40] | 8 | 29 | 58 | Asia | CS | Adult group | DXA |
| He,2009[41] | 8 | 18 | 49 | Asia | CS | Premenopausal group | DXA |
| Cervera, 1999[42] | 75 | NA | 1000 | Europe | Cohort Study | Adult group | Unspecified |
| Ceccarelli, 2023[43] | 33 | 29 | 86 | Europe | CS | Adult group | DXA |
| Carli, 2016[44] | 52 | 45 | 186 | Europe | CS | Adult group | DXA |
| F. PONS,1995[45] | 5 | NA | 28 | Europe | CS | Premenopausal group | DXA |
| Shogo Banno,2002[46] | 12 | 30 | 60 | Asia | CS | Premenopausal group | DXA |
| Becker,2001[47] | 7 | 17 | 64 | Europe | CS | Adult group | DXA |
| Zhu, 2021[48] | 27 | NA | 106 | Asia | CC | Premenopausal group | DXA |
| Jacobs, 2013[49] | 8 | 50 | 126 | Europe | Cohort Study | Adult group | DXA |
| Mok, 2022[50] | 62 | NA | 229 | Asia | Cohort Study | Adult group | DXA |
| Lai,2015[51] | 3 | 13 | 25 | Asia | CS | Postmenopausal females plus males aged > 50 years | DXA |
| Ding,2002[52] | 8 | NA | 46 | Asia | CS | Premenopausal group | DXA |
| Liang,2006[53] | 21 | 61 | 142 | Asia | CS | Premenopausal group | DXA |
| Shen,2004[54] | 15 |  | 74 | Asia | CS | Adult group | DXA |
| Li,2022[55] | 16 | 41 | 92 | Asia | CS | Adult group | DXA |
| Ye,2013[56] | 8 | 6 | 42 | Asia | CS | Adult group | DXA |
| Zhu,2000[57] | 8 | 39 | 100 | Asia | CS | Premenopausal group | DXA |
| Liu,2000[58] | 4 | 22 | 50 | Asia | CS | Premenopausal group | DXA |
| Hou,2015[59] | 96 | NA | 261 | Asia | CS | Adult group | Unspecified |
| Luo,2014[60] | 11 | 21 | 128 | Asia | CS | Premenopausal group | DXA |
| Wiebe, 2025[61] | 21 | NA | 110 | Europe | CS | Adult group | DXA |

**Supplementary Table S2：The prevalence of SLE in patients with osteoporosis across different anatomical sites.**

| Author | OP/lumbar | Number of SLE cases | OPe/lumbar | Number of SLE cases | OP/Femoral Neck | Number of SLE cases | OPe/Femoral Neck | Number of SLE cases | OP/Total Hip | Number of SLE cases | OPe/Total Hip | Number of SLE cases | area | Study type | Participants | Diagnosis |
| --- | --- | --- | --- | --- | --- | --- | --- | --- | --- | --- | --- | --- | --- | --- | --- | --- |
| Zhu,2014[62] | 19 | 125 | 48 | 125 | 10 | 123 | 57 | 123 | 10 | 122 | 44 | 122 | Asia​ | Cohort Study | Females only | DXA |
| Wiebe,2025[61] | 14 | 108 | 42 | 108 | 7 | 109 | 51 | 109 | 9 | 109 | 46 | 109 | Europe | cs | Both sexes | DXA |
| Salman-Monte,2015[63] | 2 | 66 | 24 | 66 | 6 | 67 | 31 | 67 | 3 | 67 | 19 | 67 | Europe | cs | Females only | DXA |
| Lee,2012[24] | 10 | 89 | 31 | 89 | 5 | 89 | 40 | 89 | 3 | 89 | 30 | 89 | North America | cs | Post-menopausal women | DXA |
| Gilboe,2000[64] | 7 | 75 | 21 | 75 | 5 | 75 | 26 | 75 | 3 | 75 | 32 | 75 | Europe | cs | Both sexes | DXA |

# **Supplementary Table S3：Factors Influencing Osteoporosis and Low Bone Mineral Density in Systemic Lupus Erythematosus**

| Author | Number of OP cases | Number of SLE cases | Area | Study type | participants | Diagnosis | Risk Factors for Osteoporosis |
| --- | --- | --- | --- | --- | --- | --- | --- |
| Davidson, 2018[65] | 118 | NA | North America | CC | Both sexes | Clinical Event | ①②③④⑤ |
| Eviatar, 2024[66] | 320 | 1073 | Asia | CS | Both sexes | Unspecified | ①②③④ |
| Sun, 2022[67] | NA | 125 | Asia | CS | Both sexes | DXA | ①②③④ |
| Yee, 2014[13] | 25 | 242 | Europe | CS | Adult group | DXA | ② |
| Gamal, 2023[39] | 80 | 902 | Africa | Cohort Study | Adult group | Unspecified | ②③④⑤ |
| Li, 2022[55] | 16 | 92 | Asia | CS | Adult group | DXA | ② |
| Zhong, 2023[68] | NA | 105 | Asia | CC | Both sexes | DXA | ②③⑤ |
| Wiebe, 2025[61] | 45 | 110 | Europe | CS | Both sexes | DXA | ② |
| Jung, 2020[25] | 51 | 155 | Asia | CS | Adult group | DXA | ③⑤ |
| Chen, 2018[69] | 1769 | 11288 | Asia | Cohort Study | Adult group | Clinical Event | ③ |
| Shogo Banno, 2002[46] | 12 | 60 | Asia | CS | Premenopausal group | DXA | ③ |
| Author | Number of low bone density cases | Number of SLE cases | Area | Study type | Participants | Diagnosis | Risk Factors for low bone density |
| Bonfá, 2015[70] | 51 | 211 | South America | CS | Pre-menopausal women | DXA | ① |
| Yee, 2014[13] | 123 | 242 | Europe | CS | Adult group | DXA | ① |
| Luo, 2014[60] | 32 | 128 | Asia | CS | Premenopausal group | DXA | ① |
| Cramarossa, 2017[31] | 90 | 286 | North America | CS | Adult group | DXA | ① |
| Lakshminarayanan, 2001[71] | 56 | 89 | North America | CS | Females only | DXA | ② |
| Furukawa, 2011[40] | 37 | 58 | Asia | CS | Adult group | DXA | ② |
| Li, 2022[55] | 57 | 92 | Asia | CS | Adult group | DXA | ② |

Risk Factors for Osteoporosis:①sex②age③glucocorticoid④SLEDAI⑤disease duration

Risk Factors for low bone density:①glucocorticoid②menopausal status

CS:Cross-Sectional Study(AHRQ)

CC:Case-Control Study(NOS)

Cohort Study:（NOS）

**Supplementary Table S4:Newcastle-Ottawa Quality Assessment Form for Cohort Studies**

Note: A study can be given a maximum of one star for each numbered item within the Selection and Outcome categories. A

maximum of two stars can be given for Comparability.

**Selection**

1) Representativeness of the exposed cohort

a) Truly representative ***(one star)***

b) Somewhat representative ***(one star)***

c) Selected group

d) No description of the derivation of the cohort

2) Selection of the non-exposed cohort

a) Drawn from the same community as the exposed cohort ***(one star)***

b) Drawn from a different source

c) No description of the derivation of the non exposed cohort

3) Ascertainment of exposure

a) Secure record (e.g., surgical record) ***(one star)***

b) Structured interview ***(one star)***

c) Written self report

d) No description

e) Other

4) Demonstration that outcome of interest was not present at start of study

a) Yes ***(one star)***

b) No

**Comparability**

1) Comparability of cohorts on the basis of the design or analysis controlled for confounders

a) The study controls for age, sex and marital status ***(one star)***

b) Study controls for other factors (list) _________________________________ ***(one star)***

c) Cohorts are not comparable on the basis of the design or analysis controlled for confounders

**Outcome**

1) Assessment of outcome

a) Independent blind assessment ***(one star)***

b) Record linkage ***(one star)***

c) Self report

d) No description

e) Other

2) Was follow-up long enough for outcomes to occur

a) Yes ***(one star)***

b) No

Indicate the median duration of follow-up and a brief rationale for the assessment above:____________________

3) Adequacy of follow-up of cohorts

a) Complete follow up- all subject accounted for ***(one star)***

b) Subjects lost to follow up unlikely to introduce bias- number lost less than or equal to 20% or description of those lost

suggested no different from those followed. ***(one star)***

c) Follow up rate less than 80% and no description of those lost

d) No statement

E-17Thresholds for converting the Newcastle-Ottawa scales to AHRQ standards (good, fair, and

poor):

**Good quality:**3 or 4 stars in selection domain AND 1 or 2 stars in comparability domain AND 2

or 3 stars in outcome/exposure domain

**Fair quality:**2 stars in selection domain AND 1 or 2 stars in comparability domain AND 2 or 3

stars in outcome/exposure domain

**Poor quality:**0 or 1 star in selection domain OR 0 stars in comparability domain OR 0 or 1 stars

in outcome/exposure domain

The Newcastle-Ottawa Quality Assessment scale to evalute risk of bias in three domains for cohort study and Case-Control Study.

| Studies | Selection | Comparability | Outcome | Total score |
| --- | --- | --- | --- | --- |
| Zhu, 2021[48] | 4 | 2 | 2 | 8 |
| Davidson, 2018[65] | 4 | 2 | 3 | 9 |
| Zhong, 2023[68] | 3 | 2 | 2 | 7 |
| García-Carrasco, 2017[28] | 4 | 2 | 2 | 8 |
| Boyanov, 2003[33] | 3 | 2 | 3 | 8 |
| Gamal, 2023[39] | 3 | 2 | 2 | 7 |
| Cervera, 1999[42] | 3 | 1 | 3 | 7 |
| Jacobs, 2013[49] | 4 | 2 | 3 | 9 |
| Mok, 2022[50] | 3 | 1 | 2 | 6 |
| Zhu,2014[62] | 4 | 2 | 3 | 9 |
| Chen, 2018[69] | 4 | 2 | 2 | 8 |

**Supplementary Table S5:Cross-Sectional/Prevalence Study Quality**

| Item | Yes | No | Unclear |
| --- | --- | --- | --- |
| 1) Define the source of information (survey, record review) |  |  |  |
| 2) List inclusion and exclusion criteria for exposed and unexposed subjects (cases and controls) or refer to previous publications |  |  |  |
| 3) Indicate time period used for identifying patients |  |  |  |
| 4) Indicate whether or not subjects were consecutive if not population-based |  |  |  |
| 5) Indicate if evaluators of subjective components of study were masked to other aspects of the status of the participants |  |  |  |
| 6) Describe any assessments undertaken for quality assurance purposes (e.g., test/retest of primary outcome measurements) |  |  |  |
| 7) Explain any patient exclusions from analysis |  |  |  |
| 8) Describe how confounding was assessed and/or controlled. |  |  |  |
| 9) If applicable, explain how missing data were handled in the analysis |  |  |  |
| 10) Summarize patient response rates and completeness of data collection |  |  |  |
| 11) Clarify what follow-up, if any, was expected and the percentage of patients for which incomplete data or follow-up was obtained |  |  |  |

| Studies | ① | ② | ③ | ④ | ⑤ | ⑥ | ⑦ | ⑧ | ⑨ | ⑩ | ⑪ | Total score |
| --- | --- | --- | --- | --- | --- | --- | --- | --- | --- | --- | --- | --- |
| Yee, 2014[13] | Y | Y | Y | Y | NC | N | Y | Y | NC | Y | N | 7 |
| Yeap, 2009[14] | Y | Y | N | NC | NC | Y | Y | NC | N | N | N | 4 |
| Toloza, 2010[15] | Y | Y | Y | NC | NC | N | Y | Y | N | N | N | 5 |
| Tang, 2012[16] | Y | Y | Y | Y | NC | Y | Y | Y | N | N | N | 7 |
| Sun, 2015[17] | Y | Y | Y | NC | NC | Y | Y | N | N | N | N | 5 |
| Souto, 2012[18] | Y | Y | Y | Y | NC | Y | Y | Y | N | N | N | 7 |
| Pineau, 2004[19] | Y | Y | Y | N | NC | N | Y | Y | N | N | N | 5 |
| Mendoza-Pinto, 2009（2）[20] | Y | Y | Y | NC | Y | Y | Y | Y | N | N | N | 7 |
| Mendoza-Pinto, 2009（1）[21] | Y | Y | Y | NC | NC | Y | Y | Y | N | N | N | 6 |
| Li, 2010[22] | Y | Y | N | N | NC | Y | Y | Y | N | N | N | 5 |
| Li, 2009[23] | Y | Y | N | Y | NC | Y | Y | Y | N | Y | N | 6 |
| Lee, 2012[24] | Y | Y | Y | NC | NC | Y | Y | Y | N | Y | N | 7 |
| Jung, 2020[25] | Y | Y | Y | NC | NC | Y | Y | Y | Y | N | N | 7 |
| Hansen, 2014[26] | Y | Y | Y | N | NC | Y | Y | Y | N | N | N | 6 |
| Guo, 2017[27] | Y | Y | Y | NC | NC | Y | Y | Y | N | N | N | 6 |
| Furukawa, 2013[29] | Y | Y | Y | Y | NC | Y | Y | Y | Y | Y | N | 9 |
| Fischer-Betz, 2005[30] | Y | Y | Y | Y | N | Y | Y | N | N | Y | N | 7 |
| Cramarossa, 2017[31] | Y | Y | Y | NC | Y | Y | Y | Y | N | Y | N | 8 |
| Bultink, 2005[32] | Y | NC | Y | Y | NC | Y | Y | Y | N | N | N | 6 |
| Chong, 2007[34] | Y | Y | Y | NC | N | Y | Y | Y | N | N | N | 6 |
| Almehed, 2007[35] | Y | NC | Y | NC | N | Y | Y | Y | N | Y | N | 6 |
| Correa-Rodríguez, 2021[36] | N | Y | N | Y | N | N | Y | Y | N | N | N | 4 |
| Shevchuk, 2020[37] | Y | Y | N | NC | N | Y | Y | Y | N | N | N | 5 |
| Korczowska, 2003[38] | Y | N | N | NC | N | N | N | N | N | N | N | 1 |
| Furukawa, 2011[40] | Y | NC | Y | Y | N | Y | N | Y | N | N | N | 5 |
| He,2009[41] | Y | Y | N | NC | N | N | N | Y | N | N | N | 3 |
| Ceccarelli, 2023[43] | Y | Y | N | NC | N | N | N | N | N | N | N | 2 |
| Carli, 2016[44] | Y | N | N | NC | N | N | N | Y | N | N | N | 2 |
| F. PONS, 1995[45] | Y | Y | N | NC | N | N | N | N | N | N | N | 2 |
| Shogo Banno, 2002[46] | Y | Y | N | Y | N | Y | Y | Y | N | N | N | 6 |
| Becker, 2001[47] | Y | Y | Y | NC | N | N | Y | Y | N | N | N | 5 |
| Lai, 2015[51] | Y | Y | N | Y | N | N | N | Y | N | N | N | 4 |
| Ding, 2002[52] | Y | Y | Y | NC | N | N | N | N | N | N | N | 3 |
| Liang, 2006[53] | Y | Y | N | NC | N | N | N | N | N | N | N | 2 |
| Shen, 2004[54] | Y | Y | N | NC | N | N | N | Y | N | N | N | 3 |
| Li, 2022[55] | Y | Y | Y | NC | N | N | N | Y | N | N | N | 4 |
| Ye, 2013[56] | Y | Y | Y | NC | N | N | N | N | N | N | N | 3 |
| Zhu, 2000[57] | Y | Y | Y | NC | N | N | N | N | N | N | N | 3 |
| Liu, 2000[58] | Y | Y | Y | NC | N | N | N | N | N | N | N | 3 |
| Hou, 2015[59] | Y | Y | Y | NC | N | Y | Y | Y | N | N | N | 6 |
| Luo, 2014[60] | Y | Y | Y | NC | N | Y | N | Y | N | N | N | 5 |
| Wiebe, 2025[61] | Y | Y | Y | Y | N | Y | Y | Y | Y | N | N | 8 |
| Salman-Monte, 2015[63] | Y | Y | Y | Y | N | N | N | N | N | N | N | 4 |
| Gilboe, 2000[64] | Y | Y | N | NC | N | Y | N | Y | N | N | N | 4 |
| Eviatar, 2024[66] | Y | Y | Y | NC | N | N | NC | Y | N | N | N | 4 |
| Sun, 2022[67] | Y | Y | Y | NC | N | N | N | Y | N | N | N | 4 |
| Bonfá, 2015[70] | Y | Y | Y | NC | N | NC | N | Y | N | N | N | 4 |
| Lakshminarayanan, 2001[71] | Y | N | Y | Y | N | Y | N | Y | N | Y | N | 6 |

**Supplementary Table S6：Prevalence of osteoporosis in the lumbar spine**

| Subgroup Name | Number of people with osteoporosis | Total Sample Size (n) | Detection rates/(95%CI)(%) | P Value | I2(%) | Effect Model | Egger |
| --- | --- | --- | --- | --- | --- | --- | --- |
| Prevalence of lumbar spine disorders | 52 | 463 | 10.0（5.2，14.9） | P＜0.01 | 70.7% | random | 0.13 |
| Area | | | | | | | |
| Europe | 23 | 249 | 8.1（1.8，14.3） | 0.012 | 73.0% | random |  |
| Asia | 19 | 125 | 15.2（9.4，22.7） | P＜0.01 | / | random |  |
| North America | 10 | 89 | 11.2（5.5，19.7） | P＜0.01 | / | random |  |

**Supplementary Table S7：Prevalence of osteoporosis in the femoral neck**

| Subgroup Name | Number of people with osteoporosis | Total Sample Size (n) | Detection rates/(95%CI)(%) | P Value | I2(%) | Effect  Model | Egger |
| --- | --- | --- | --- | --- | --- | --- | --- |
| Prevalence of femoral neck fractures | 33 | 463 | 7.0（4.6，9.3） | P＜0.01 | 0% | fixed | 0.617 |
| Area | | | | | | | |
| Europe | 18 | 249 | 7.0（3.9，10.2） | P＜0.01 | 0% | fixed |  |
| Asia | 10 | 125 | 8.1（3.3，13.0） | P＜0.01 | / | fixed |  |
| North America | 5 | 89 | 5.6（0.8，10.4） | 0.021 | / | fixed |  |

**Supplementary Table S8：Prevalence of osteoporosis in the total hip**

| Subgroup Name | Number of people with osteoporosis | Total Sample Size (n) | Detection rates/(95%CI)(%) | P Value | I2(%) | Effect Model | Egger |
| --- | --- | --- | --- | --- | --- | --- | --- |
| Prevalence of total hip conditions | 28 | 463 | 5.3（3.3，7.3） | P＜0.01 | 0% | fixed | 0.298 |
| Area | | | | | | | |
| Europe | 15 | 249 | 5.4（2.6，8.2） | P＜0.01 | 0% | fixed |  |
| Asia | 10 | 125 | 8.2（4.0，14.6） | P＜0.01 | / | fixed |  |
| North America | 3 | 89 | 3.4（0.7，9.5） | 0.078 | / | fixed |  |

**Supplementary Table S9：Prevalence of osteopenia in the lumbar spine**

| Subgroup Name | Number of individuals with reduced bone mass | Total Sample  Size(n) | Detection rates/(95%CI)(%) | P Value | I2(%) | Effect  Model | Egger |
| --- | --- | --- | --- | --- | --- | --- | --- |
| Prevalence of lumbar spine disorders | 166 | 463 | 35.6（31.3，40.0） | P＜0.01 | 0% | fixed | 0.078 |
| Area | | | | | | | |
| Europe | 87 | 249 | 34.6（28.7，40.5） | P＜0.01 | 21.4% | fixed |  |
| Asia | 48 | 125 | 38.4（29.9，46.9） | P＜0.01 | / | fixed |  |
| North America | 31 | 89 | 34.8（24.9，44.7） | P＜0.01 | / | fixed |  |

**Supplementary Table S10：Prevalence of osteopenia in the femoral neck**

| Subgroup Name | Number of individuals with reduced bone mass | Total Sample Size(n) | Detection rates/(95%CI)(%) | P Value | I2(%) | Effect Model | Egger |
| --- | --- | --- | --- | --- | --- | --- | --- |
| Prevalence of femoral neck fractures | 301 | 463 | 44.1（39.6，48.6） | P＜0.01 | 0% | fixed | 0.078 |
| Area | | | | | | | |
| Europe | 108 | 249 | 42.8（36.7，48.9） | P＜0.01 | 37.7% | fixed |  |
| Asia | 80 | 125 | 46.3（37.5，55.2） | P＜0.01 | / | fixed |  |
| North America | 70 | 89 | 44.9（34.6，55.3） | P＜0.01 | / | fixed |  |

**Supplementary Table S11：Prevalence of osteopenia in the total hip**

| Subgroup Name | Number of individuals with reduced bone mass | Total SampleSize(n) | Detection rates/(95%CI)(%) | P Value | I2(%) | Effect Model | Egger |
| --- | --- | --- | --- | --- | --- | --- | --- |
| Prevalence of total hip conditions | 333 | 463 | 36.7（32.3，41.1） | P＜0.01 | 21.8% | fixed | 0.243 |
| Area | | | | | | | |
| Europe | 160 | 249 | 38.1（32.2，44.1） | P＜0.01 | 55.8% | fixed |  |
| Asia | 44 | 125 | 36.1（27.5，44.6） | P＜0.01 | / | fixed |  |
| North America | 129 | 89 | 33.7（23.9，43.5） | P＜0.01 | / | fixed |  |

**Supplementary Figure:Further investigation into the high heterogeneity of the ‘other diagnostic criteria’ subgroup**

**Supplementary Figure S1：Meta-regression of geographic region on the prevalence of osteoporosis in the “Other Diagnostic Criteria” subgroup**


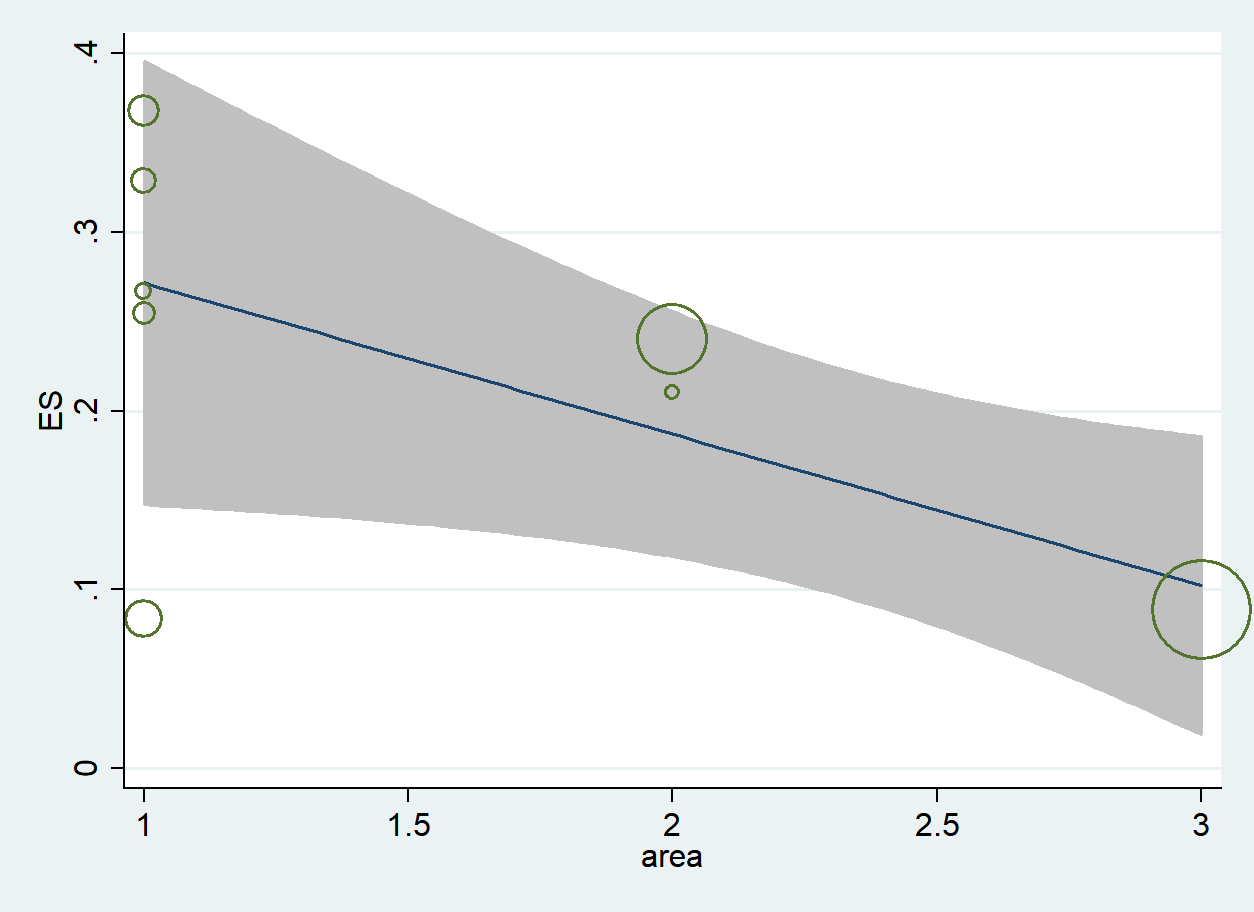


**Supplementary Figure S2：Meta-regression of participant type (e.g., pre-/post-menopausal) on the prevalence of osteoporosis in the “Other Diagnostic Criteria” subgroup**


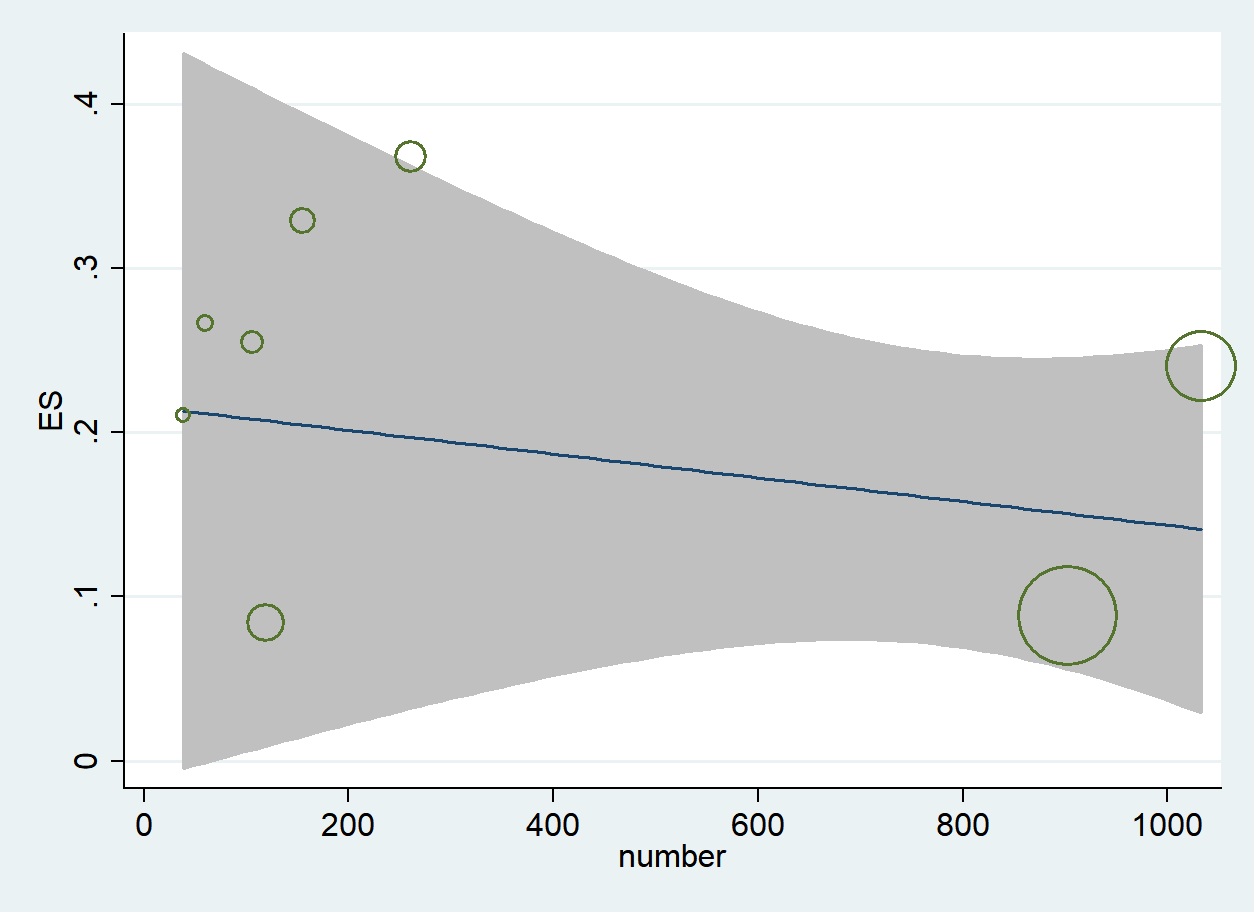


**Supplementary Figure S3：** **Meta-regression of participant type (e.g., pre-/post-menopausal) on the prevalence of osteoporosis in the “Other Diagnostic Criteria” subgroup**


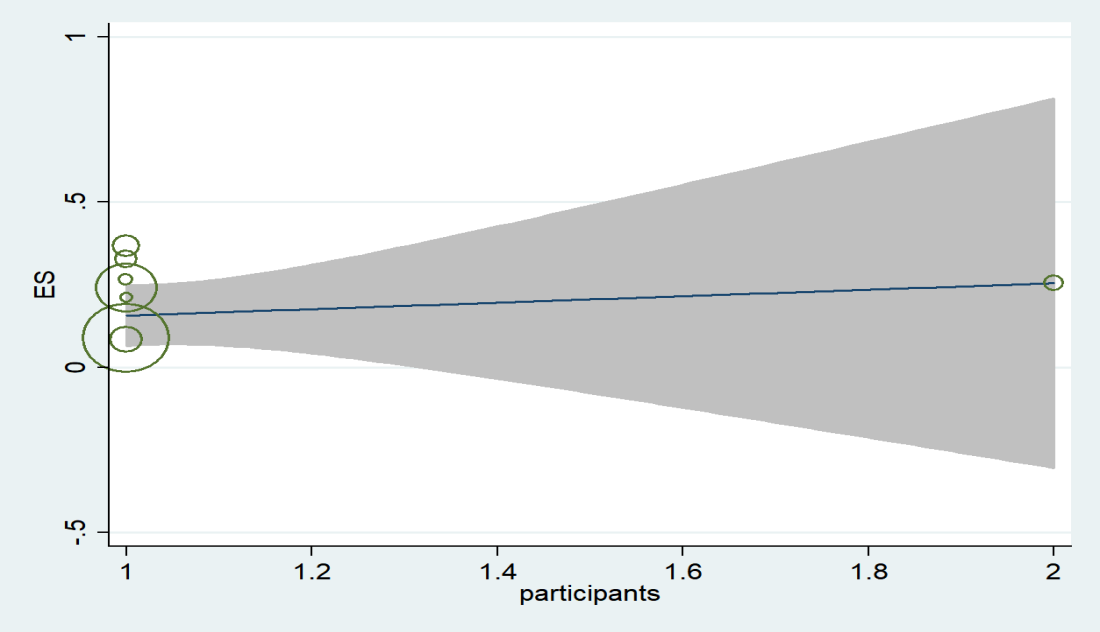


**Supplementary Figure S4：** **Meta-regression of sex composition on the prevalence of osteoporosis in the “Other Diagnostic Criteria” subgroup**


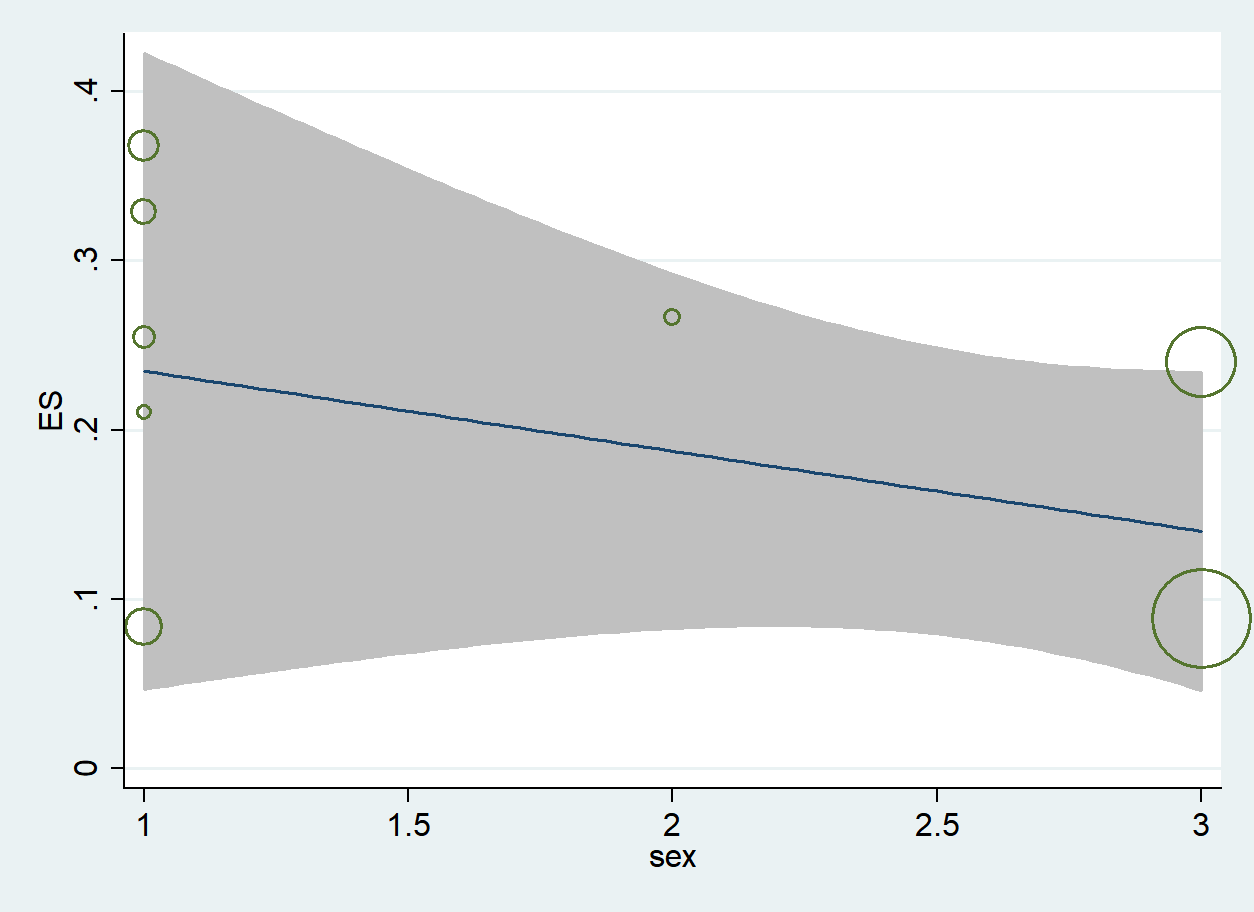


**Supplementary Figure:Total and site-specific prevalence of osteoporosis in patients with systemic lupus erythematosus (lumbar spine, femoral neck, total hip).**

**Supplementary Figure S5:Funnel plot: Overall Prevalence of Osteoporosis in Systemic Lupus Erythematosus.**

**Supplementary Figure S6:Forest plot: Prevalence of lumbar osteoporosis in systemic lupus erythematosus, by study region.**


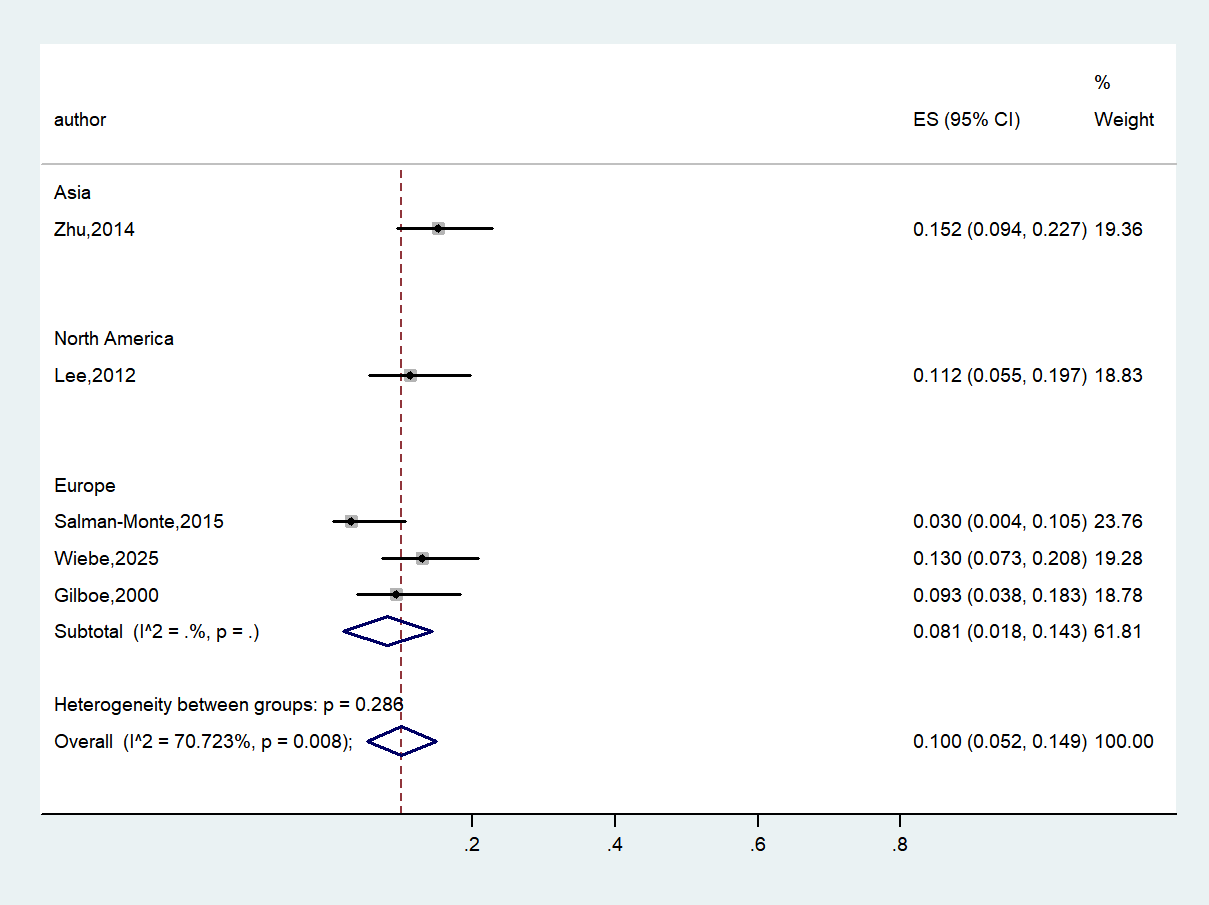


**Supplementary Figure S7:Forest plot: Prevalence of osteoporosis at the femoral neck in systemic lupus erythematosus, by study region.**

**Supplementary Figure S8:Forest plot: Prevalence of osteoporosis at the total hip in systemic lupus erythematosus, by study region.**


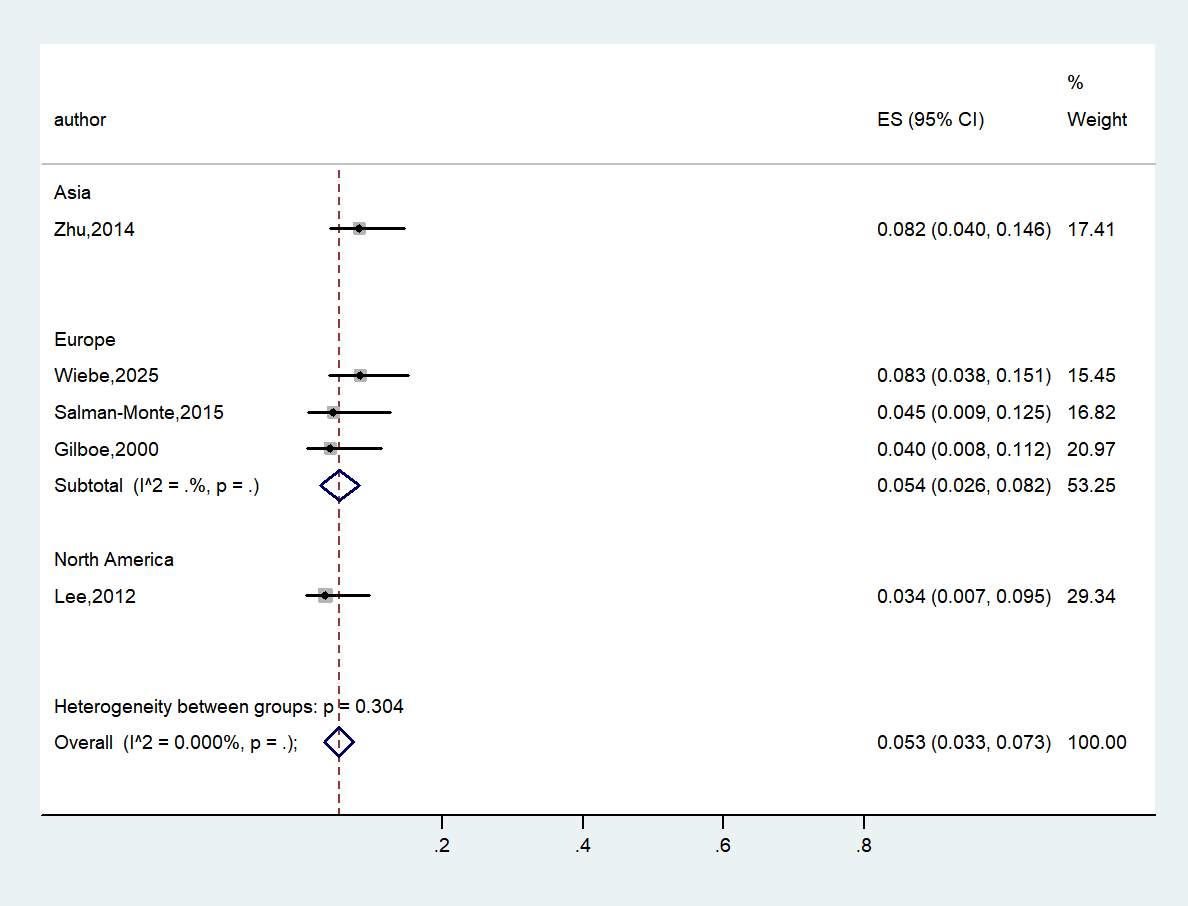


**Supplementary Figure S9:Sensitivity analysis: Prevalence of osteoporosis in the lumbar spine in systemic lupus erythematosus.**

**Supplementary Figure S10:Funnel plot: Prevalence of lumbar osteoporosis in systemic lupus erythematosus.**

**Supplementary Figure S11:Funnel plot: Prevalence of osteoporosis at the femoral neck in systemic lupus erythematosus.**

**Supplementary Figure S12:Funnel plot: Prevalence of osteoporosis at the total hip in systemic lupus erythematosus.**

**Supplementary Figure S13:Sensitivity analysis: Prevalence of osteopenia in Systemic Lupus Erythematosus.**

**Supplementary Figure S14:Funnel plot: Prevalence of Osteopenia in Systemic Lupus Erythematosus.**

**Supplementary Figure S15:Forest plot: Prevalence of osteopenia at the lumbar spine in systemic lupus erythematosus, by study region.**

**Supplementary Figure S16:Forest plot: Prevalence of osteopenia at the femoral neck in systemic lupus erythematosus, by study region.**

**Supplementary Figure S17:Forest plot: Prevalence of osteopenia at the total hip in systemic lupus erythematosus, by study region.**

**Supplementary Figure S18:Funnel plot: Prevalence of osteopenia at the lumbar spine in systemic lupus erythematosus.**

**Supplementary Figure S19:Funnel plot: Prevalence of osteopenia at the femoral neck in systemic lupus erythematosus.**

**Supplementary Figure S20:Funnel plot: Prevalence of osteopenia at the total hip in systemic lupus erythematosus.**

**Supplementary figure: Factors influencing prevalence**

**Supplementary Figure S21:Forest plot:Risk factors for osteoporosis in systemic lupus erythematosus,by sex.**

**Supplementary Figure S22:Sensitivity analysis:Risk factors for osteoporosis in systemic lupus erythematosus, by sex.**

**Supplementary Figure S23:Funnel plot: Risk factors for osteoporosis in systemic lupus erythematosus, by sex.**

**Supplementary Figure S24:Forest plot:Risk factors for osteoporosis in systemic lupus erythematosus,by age.**

**Supplementary Figure S25:Forest Plot: Risk Factors for Osteoporosis in Systemic Lupus Erythematosus, Stratified by Age and Further Subgrouped According to Geographic Region.**

**Supplementary Figure S26:Sensitivity analysis:Risk factors for osteoporosis in systemic lupus erythematosus, by age.**

**Supplementary Figure S27:Funnel plot: Risk factors for osteoporosis in systemic lupus erythematosus, by age.**

**Supplementary Figure S28:Forest Plot: Risk Factors for Osteoporosis in Systemic Lupus Erythematosus, Stratified by glucocorticoid utilization rate and Further Subgrouped According to Geographic Region.**

**Supplementary Figure S29:Forest Plot: Risk Factors for Osteoporosis in Systemic Lupus Erythematosus, Stratified by glucocorticoid utilization rate and Further Subgrouped by Study Population Characteristics.**

**Supplementary Figure S30:Forest Plot: Risk Factors for Osteoporosis in Systemic Lupus Erythematosus, Stratified by glucocorticoid dosage.**

**Supplementary Figure S31:Sensitivity analysis:Risk factors for osteoporosis in systemic lupus erythematosus, by glucocorticoid .**

**Supplementary Figure S32:Funnel plot: Risk factors for osteoporosis in systemic lupus erythematosus, by glucocorticoid.**

**Supplementary Figure S33:Forest Plot: Risk factors for osteoporosis in systemic lupus erythematosus, by disease duration and Further Subgrouped According to Geographic Region.**

**Supplementary Figure S34:Sensitivity analysis: Risk factors for osteoporosis in systemic lupus erythematosus, by disease duration.**

**Supplementary Figure S35:Funnel plot: Risk factors for osteoporosis in systemic lupus erythematosus, by disease duration.**

**Supplementary Figure S36:Forest Plot: Risk factors for osteoporosis in systemic lupus erythematosus, by SLE Disease Activity Index (SLEDAI) and Further Subgrouped According to Geographic Region.**

**Supplementary Figure S37:Sensitivity analysis: Risk factors for osteoporosis in systemic lupus erythematosus, by SLE Disease Activity Index (SLEDAI).**

**Supplementary Figure S38:Funnel plot: Risk factors for osteoporosis in systemic lupus erythematosus, by SLE Disease Activity Index (SLEDAI).**

**Supplementary Figure S39:Forest plot:Risk factors for Low Bone Mineral Density in systemic lupus erythematosus,by by glucocorticoid utilization rate and Further Subgrouped According to Geographic Region.**

**Supplementary Figure S40:Sensitivity analysis: Risk factors for Low Bone Mineral Density in systemic lupus erythematosus,by glucocorticoid utilization rate.**

**Supplementary Figure S41:Funnel plot: Risk factors for Low Bone Mineral Density in systemic lupus erythematosus,by glucocorticoid utilization rate.**

**Supplementary Figure S42:Forest plot: Risk factors for Low Bone Mineral Density in systemic lupus erythematosus, by menopausal status.**

**Supplementary Figure S43:Funnel plot: Risk factors for Low Bone Mineral Density in systemic lupus erythematosus, by menopausal status.**
